# Effect of the closed‐loop hippocampal low‐frequency stimulation on seizure severity, learning, and memory in pilocarpine epilepsy rat model

**DOI:** 10.1111/cns.14656

**Published:** 2024-03-04

**Authors:** Meysam Zare, Mahmoud Rezaei, Milad Nazari, Nastaran Kosarmadar, Mona Faraz, Victoria Barkley, Amir Shojaei, Mohammad Reza Raoufy, Javad Mirnajafi‐Zadeh

**Affiliations:** ^1^ Department of Physiology, Faculty of Medical Sciences Tarbiat Modares University Tehran Iran; ^2^ Department of Technology, Electrical Engineering Sharif University Tehran Iran; ^3^ Department of Anesthesia and Pain Management, Toronto General Hospital University Health Network Toronto Ontario Canada; ^4^ Institute for Brain Sciences and Cognition Tarbiat Modares University Tehran Iran

**Keywords:** closed‐loop system, deep brain stimulation, medial prefrontal cortex, spatial memory, ventral hippocampus

## Abstract

**Aims:**

In this study, the anticonvulsant action of closed‐loop, low‐frequency deep brain stimulation (DBS) was investigated. In addition, the changes in brain rhythms and functional connectivity of the hippocampus and prefrontal cortex were evaluated.

**Methods:**

Epilepsy was induced by pilocarpine in male Wistar rats. After the chronic phase, a tripolar electrode was implanted in the right ventral hippocampus and a monopolar electrode in medial prefrontal cortex (mPFC). Subjects' spontaneous seizure behaviors were observed in continuous video recording, while the local field potentials (LFPs) were recorded simultaneously. In addition, spatial memory was evaluated by the Barnes maze test.

**Results:**

Applying hippocampal DBS, immediately after seizure detection in epileptic animals, reduced their seizure severity and duration, and improved their performance in Barnes maze test. DBS reduced the increment in power of delta, theta, and gamma waves in pre‐ictal, ictal, and post‐ictal periods. Meanwhile, DBS increased the post‐ictal‐to‐pre‐ictal ratio of theta band. DBS decreased delta and increased theta coherences, and also increased the post‐ictal‐to‐pre‐ictal ratio of coherence. In addition, DBS increased the hippocampal‐mPFC coupling in pre‐ictal period and decreased the coupling in the ictal and post‐ictal periods.

**Conclusion:**

Applying closed‐loop, low‐frequency DBS at seizure onset reduced seizure severity and improved memory. In addition, the changes in power, coherence, and coupling of the LFP oscillations in the hippocampus and mPFC demonstrate low‐frequency DBS efficacy as an antiepileptic treatment, returning LFPs to a seemingly non‐seizure state in subjects that received DBS.

## Graphical abstract

Applying closed‐loop low‐frequency DBS, immediately after seizure detection, reduced the seizure severity, improved memory, and restored the changes in power, coherence, and coupling of the LFP oscillations in the hippocampus and medial prefrontal cortex. These effects assigned the low‐frequency DBS as an effective antiepileptic treatment.

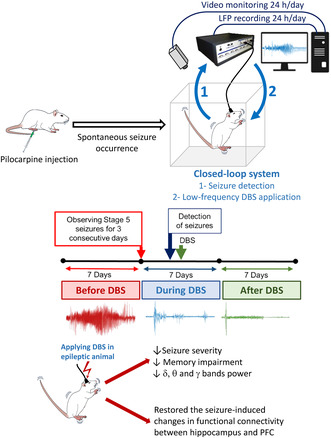

## 1 INTRODUCTION

Temporal lobe epilepsy (TLE) is the most common type of medically refractory epilepsy in adults.^1^ TLE seizures are associated with abnormal activity, including hypersynchrony of large populations of neurons.^2^ These abnormal activities in the epileptic brain are accompanied by alterations in normal neurophysiological brain rhythms, including delta, theta, and gamma oscillations, and sharp‐wave ripples.^3^

Epileptic seizures may disrupt the functional connectivity and neuronal coherence among different brain areas, including the hippocampus and medial prefrontal cortex (mPFC). A human functional magnetic resonance imaging study showed significant changes in the connectivity between the hippocampus and mPFC in epilepsy patients.^4^ A reduction in hippocampus and mPFC coherence was also observed in rats with epilepsy.^5^ Normal neuronal activity and synchronization in the hippocampus and mPFC are necessary for cognitive functions.^6^, ^7^ Furthermore, the connection between the hippocampus and the mPFC plays an important role in memory processes.^8^

It has long been documented that the hippocampus plays a crucial role in the formation of long‐term memory in humans^9^ and spatial memory in rodents.^10^ Severe damage to the hippocampus has been observed in TLE patients with consequent memory impairments. In addition, in the pilocarpine epilepsy model in rats, pilocarpine‐treated subjects demonstrated reduced spatial memory.^11^ The mPFC also plays a key role in higher‐level, cognitive functions.^12^ The rodent mPFC plays the same function in cognitive processes as primates' ventral‐medial and dorsal‐lateral PFC.^13^ Accordingly, the common impairments in learning and memory in TLE^14^ may somehow be related to the disrupted functional connectivity between the hippocampus and mPFC.

Deep brain stimulation (DBS) applied in the hippocampus is one treatment for drug‐resistant epilepsy.^15^ DBS is commonly administered continuously without considering the brain activity, that is, open‐loop DBS. However, it may be more effective when applied following the detection of a specific brain activity, that is, closed‐loop DBS.^16^ Different factors involve in DBS anticonvulsant action, including its target, time of application, and frequency.

The best DBS target for exerting its anticonvulsant action is under debate. However, it has been suggested that the anticonvulsant mechanism of DBS depends on its stimulated targets. One such anticonvulsant mechanism, named gating mechanism, requires applying DBS to seizure‐controlling sites, which contribute to seizure propagation; in this case, DBS stimulation inhibits the propagation of epileptiform discharges.^17^ The second anticonvulsant mechanism is related to direct stimulation and therefore inhibition of the presumable seizure foci.^18^

DBS frequency is another parameter in determining DBS effectiveness. Applying high‐frequency DBS in epilepsy patients exerts anticonvulsant action.^19^, ^20^ However, some reports show the anticonvulsant effects of low‐frequency DBS in epilepsy patients^21^, ^22^ and in animal models.^21^, ^23^ In addition, applying low‐frequency DBS following kindling stimulations can improve learning and memory.^24^, ^25^

Regarding time of application, our previous experiment showed that DBS had a better anticonvulsant action when applied at seizure onset, that is, when epileptiform activity begins, compared to DBS applied before and/or after epileptiform activity.^26^

Considering the hippocampus' role in seizure development and the hippocampus' functional connectivity with the mPFC in memory and learning, in the present study, the effects of low‐frequency DBS on seizure severity, the functional connectivity between the hippocampus and mPFC, as well as learning and memory were investigated.

## 2 MATERIALS AND METHODS

### 2.1 Animals

Male Wistar rats (200–250 g) were obtained from the Razi Institute (Karaj, Iran). Animals were maintained under the standard conditions, 22–25°C ambient temperature, experienced a daily 12 h light/dark schedule (lights on at 8:00 a.m.), and had access to food and water ad libitum. All manipulations were in line with the ethical guidelines approved by the “Ethical Committee of the Faculty of Medical Sciences, Tarbiat Modares University.”

### 2.2 Chronic seizure induction

Subjects received lithium chloride (212 mg/kg, i.p.), then pilocarpine hydrochloride (150 mg/kg, i.p.) was injected 24 h later. Methylscopolamine bromide (1 mg/kg, i.p.) was also administered 30 min before pilocarpine hydrochloride to limit the peripheral effects of pilocarpine. Diazepam (4 mg/kg) was also administered 1 h after the first observed seizure to control status epilepticus and to prevent sudden death. However, severe tonic–clonic seizures induced by pilocarpine led to a high mortality rate (about 40%) in subjects.

Recurrent seizures started at 30–60 min after the pilocarpine administration. To distinguish the occurrence of status epilepticus, subjects were monitored by video recording following pilocarpine injection. Seizure behavior/severity was assessed according to the modified Racine limbic seizure scale^27^: stage 1, twitching of jaw and facial muscles; stage 2, facial clonus that progresses to the trunk muscles; stage 3, clonic abbreviation of forelimbs; stage 4, rearing (standing on hind legs with support on paws and tail); and stage 5, rearing and falling with clonic convulsions of extremities, generalization of seizures, and progression to tonic convulsions with complete loss of posture. Only seizures with a score of 4 or more and lasting for more than 15 min were identified as status epilepticus. The majority of animals developed seizures scoring 4 or higher 30 min after pilocarpine injection. Only rats with stage 4 or 5 seizures lasting for at least 90 min were selected for experiments. Severe tonic–clonic seizures induced by pilocarpine led to a high mortality in the animals, and only 60% of subjects showed status epilepticus.

The initial acute insult was followed by a seizure‐free phase (latent) and finally, a chronic period characterized by the occurrence of spontaneous seizures, which started at 45–60 days after the first attack. The criteria for successful induction of spontaneous seizures were observed through both continuous behavioral monitoring and LFP recording. The simultaneous occurrence of behavioral convulsions and epileptiform discharges was considered successful induction of spontaneous seizure. For this purpose, subjects were placed in a clear plexiglass cage with unrestricted access to food and water, and their behavior and LFP were continuously recorded for 3 weeks. From video monitoring and LFP recordings, the average duration of seizures and frequency of seizures was determined. The number and duration of spontaneous seizures were evaluated offline.

### 2.3 Surgery

Electrode implantation was done 30–35 days after the pilocarpine hydrochloride injection. Under 100 mg/kg ketamine (10%, Alfasan, The Netherlands) and 10 mg/kg xylazine (20%, Alfasan, The Netherlands), subjects underwent stereotaxic implantation with a tripolar recording and stimulating electrode in the CA1 region of the right ventral hippocampus (coordinates: A, −6.0 mm; L, 5.5 mm; and V, 6.8 below dura) and mPFC (coordinates: A, 3.2 mm; L, 0.6 mm; and V, 3.6 below dura).^28^ The recording electrode (stainless, steel, Teflon coated, 127 μm in diameter, A.M. Systems, USA) and reference electrode (connected to skull by a miniature screw) were insulated except at their tips. All electrodes were connected to pins of a lightweight, multichannel miniature socket and fixed on the skull with dental acrylic. The subjects had at least 10 days for post‐surgery recovery. There were no complications of the electrode implantation in epileptic animals.

### 2.4 Local field potential recording and DBS application

Local field potentials (LFPs) were recorded from the hippocampal CA1 and mPFC when the freely moving animals were put in a plexiglass recording box inside a Faraday cage. Signals were filtered at 3 kHz, amplified, and digitized (at 10 kHz) using a PC‐based data acquisition system (BIODAC ES1721, Trita Health Technology CO., Tehran, Iran) and were continuously monitored and stored on disk. LFPs were recorded 24 h a day for 1 week. Simultaneously, 24 h video recordings of subjects' behavior were collected and reviewed to evaluate the occurrence of seizure attacks based on behavioral hallmarks.

DBS application was done by BIODAC ES1721 (Trita Health Technology CO., Tehran, Iran). DBS consisted of four packages at 5 min intervals; each package contained 200 monophasic square wave pulses of 0.1 ms duration at 1 Hz (see Figure S1). DBS pattern was achieved according to our preliminary experiments on the hippocampal CA1 area. To avoid the saturation of amplifier by monophasic stimulation, which may lead to loss of signal due to tissue polarization, we used a passive charge method in the stimulator. After applying a unipolar pulse, a shorting phase is used to destroy the stored charges. In this manner, the two stimulation electrodes are short circuited to discharge the charge stored in the capacitor between the electrode and the tissue. The discharge speed of this method is in the order of several milliseconds in deep brain stimulation.

### 2.5 Learning and memory assessment

The Barnes maze test was used for evaluating changes in spatial learning and memory. We have described this test in detail in our previous study.^29^ The Barnes maze consisted of an elevated (90 cm to the floor) black acrylic circular platform, 120 cm in diameter, containing 18 holes around the periphery. The holes had uniform diameter (9 cm) and appearance, but only one hole was connected to a removable black escape box (35 cm long, 11.5 cm wide, and 10.3 cm in depth). A square starting chamber (an opaque, 20 cm × 30 cm, and 15 cm high, open‐ended chamber) is used to place the rats on the platform. Four proximal visual cues are placed in the room, 50 cm away from the circular platform. The platform was cleaned using 70% ethanol at the end of each session. In the present experiment, subjects were exposed to the Barnes maze in three phases: initial training, probe test, and reversal learning.

The initial training took place over 4 days. In the training phase, the rat was placed inside the start box, in the center of the Barnes maze for 10 s. This allowed the rat to face a random direction when the start box was lifted and the trial began. The rat was allowed to explore the maze for 3 min. If a rat found the target hole and entered the escape box, the trial ended and the subject was allowed to stay in the escape cage for 1 min before being returned to the holding cage. If the subject did not find the target hole, it was picked up and manually put on the platform in the escape cage. Then, it was allowed 1 min inside the escape box before being returned to the home cage. Four trials were done on each training day at 15 min intervals.

The probe test was done on the 5th day, in which the escape box was closed and the rats were allowed to search the maze table for 3 min. The reversal learning phase was run 24 h after the probe test. In this phase, the target hole changed to the position 180 degrees from the probe day target. Training for reversal learning was done for 3 days.

### 2.6 Experimental design

Subjects were assigned to four groups: pilocarpine + DBS (Pilo+DBS; *n* = 7), pilocarpine (Pilo; *n* = 8), control (*n* = 7), and DBS (*n* = 6). Subjects in the Pilo+DBS group were epileptic rats with spontaneous lithium–pilocarpine‐induced seizures. Continuous video monitoring and LFP recording were done for 3 weeks. During the 2nd week, these subjects received closed‐loop DBS in the ventral hippocampus during online recording and analysis of LFPs. Seizure onset was determined according to the changes in LFP power as explained in a prior study.^30^ The seizure onset was detected by a closed‐loop online system in freely moving animals. Briefly, a detection algorithm based on comparing the total power was used (because the total power was increased by seizure). Windowing analysis of signal was done to calculate the short‐term power. LFP and epileptiform records were analyzed by “signal processing toolbox” in MATLAB software. The averaged power of LFP that was calculated for a long time (10 s) was considered as base value. The best detection was achieved when we used 100 consecutive epochs (each epoch equals 10 ms) of LFP for data analysis. The changes in the power were detected as seizure onset.

Similar to the Pilo+DBS group, subjects in the pilocarpine group were epileptic rats with spontaneous lithium–pilocarpine‐induced seizures. These animals underwent long‐term monitoring and LFP recording for 3 weeks. All experimental procedures were similar to the Pilo+DBS group. However, no DBS was applied.

In the control group, subjects did not receive lithium–pilocarpine and DBS. These animals were the same age as Pilo+DBS and pilocarpine groups' subjects and underwent continuous video monitoring and LFP recording for 3 weeks.

In the DBS group, the experimental procedure was completely similar to the control group; however, they received DBS during the 2nd week of video monitoring and LFP recording. The number of DBS applications in these animals was equal to mean number of DBS applied in the Pilo+DBS group.

**FIGURE 1:**
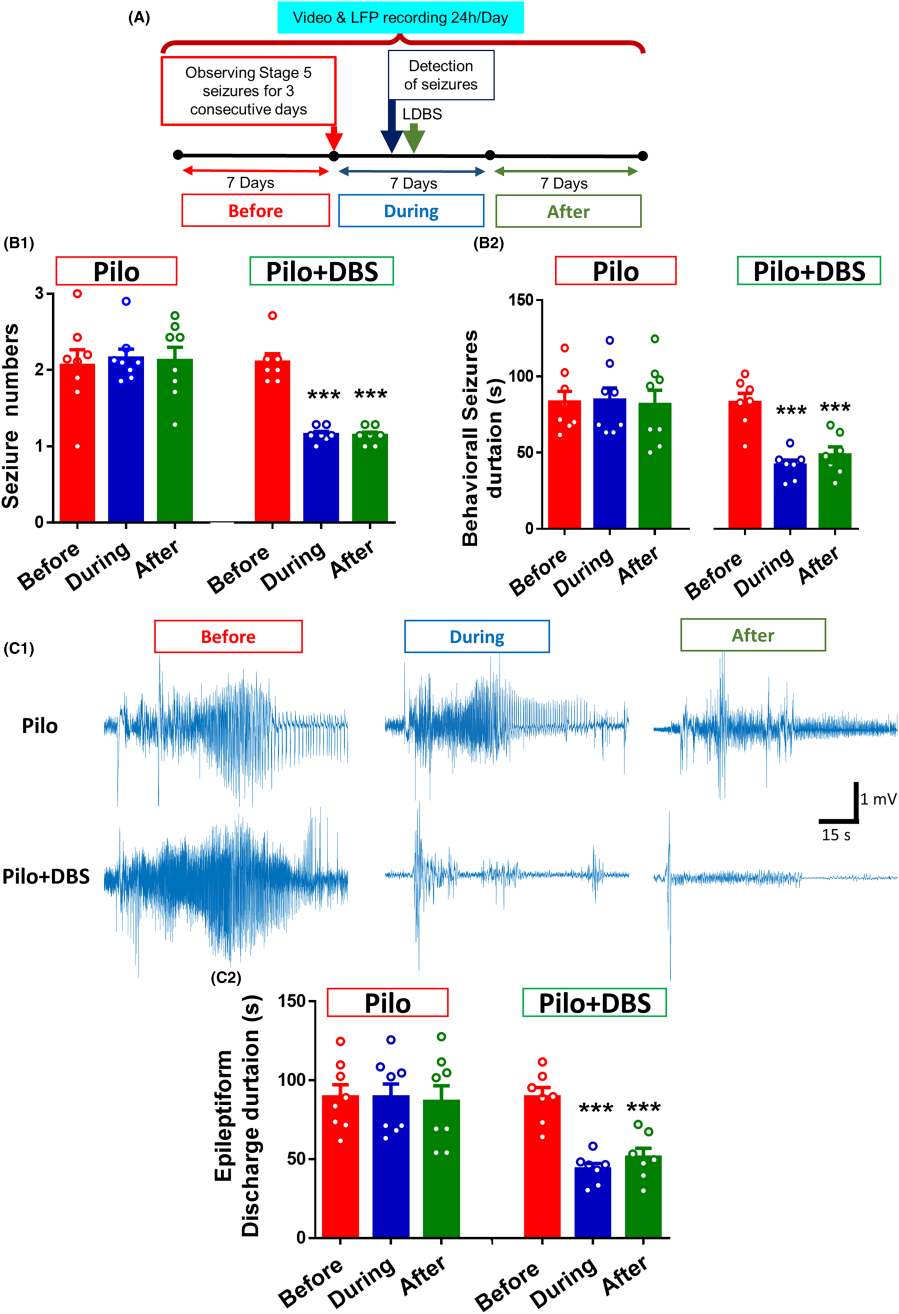
The effect of closed‐loop low‐frequency DBS on the number and duration of seizures. The time‐line diagram (A) shows the experimental periods (before, during, and after) for evaluation of seizure severity. Applying a closed‐loop DBS in epilepsy‐modeled animals reduced the number of seizures (B1) and duration of seizure behavior (B2) during and after applying DBS. Sample recordings in (C1) show the epileptiform ictal discharges in LFP recorded before, during, and after DBS application periods. Statistical analysis showed that the epileptiform discharge duration was reduced by applying a closed‐loop DBS (C2). Data show means ± SEM (*n* = 7 in the control group, *n* = 6 in the DBS group, *n* = 8 in the Pilo group, and *n* = 7 in the Pilo+DBS group). ***p < 0.001 compared to the before applying DBS period.

For LFP analysis in “Before,” “During,” and “After” spontaneous seizure periods (Figure 1A), sample data were collected from 14 seizures in the Pilo+DBS group and 24 seizures in the Pilo group. In addition, 14 sample data were collected in control and 12 sample data were collected from DBS groups at similar time scales.

### 2.7 LFP analyses

All analyses were performed with built‐in, custom‐written MATLAB (MathWorks) routines. Fieldtrip toolbox was used for removing DBS artifacts. LFP analyses were performed in the pilocarpine and Pilo+DBS groups after the third seizure in the chronic phase.

#### 2.7.1 Power spectral density and coherence spectra

We used the Welch method to calculate the power spectral density with the MATLAB pwelch function. The coherence spectrum between two regions was measured by calculating the magnitude squared coherence using the mscohere function in MATLAB. Power spectrum and coherence calculations were both performed on 30s segments of data using 6 ms Hamming windows with 90% overlap.

#### 2.7.2 Cross‐frequency coupling

Phase–amplitude coupling was calculated within and between regions by using the modulation index method described in Tort et al.^31^ We analyzed the phase coupling of the bands 1–12 Hz (0.5 Hz step) and the amplitude of the gamma band between 35 and 250 Hz. Theta phases were binned into eighteen 20° intervals and then the corresponding gamma amplitude was averaged for each bin. We calculated the phase–amplitude modulation index (MI) by deviating the experimental phase amplitude profile from a uniform distribution. A composite plot was obtained by calculating the MI between several pairs of filtered band frequencies, displaying the results in a two‐dimensional heatmap.

### 2.8 Statistical analysis

Statistical analyses were performed using GraphPad Prism software version 6.01 for Windows (CA, USA). The normality of the data distribution within each parameter was verified by Kolmogorov–Smirnov normality test. A two‐way repeated measures ANOVA followed by a Tukey's post hoc test was used to compare Barnes test data for training days. In addition, a one‐way ANOVA and Tukey's post hoc test were employed to compare parameters of the Barnes probe test and power, coherence, and phase–amplitude coupling. Data are presented as means ± SEM, and the level of significance was *p* < 0.05.

## 3 RESULTS

### 3.1 Effect of closed‐loop DBS on seizures

Epileptic animals received DBS for 7 days. A two‐way ANOVA showed a significant effect of DBS on number of seizures in the Pilo+DBS group (*F*_(2,26)_ = 13.71; *p* < 0.0001) (Figure 1A,B). A Tukey's post hoc test showed that applying DBS significantly reduced the number of seizures during and after DBS (*p* < 0.0001) (Figure 1A,B1). There was also a significant decrement in the duration of behavioral seizure (*F*_(2,26)_ = 20.96; *p* < 0.0001) and epileptiform discharges (*F*_(2,26)_ = 26.64; *p* < 0.0001) in the Pilo+DBS group during and after DBS application (Figure 1B2,C1,C2).

### 3.2 Effect of closed‐loop DBS on learning and memory

**FIGURE 2:**
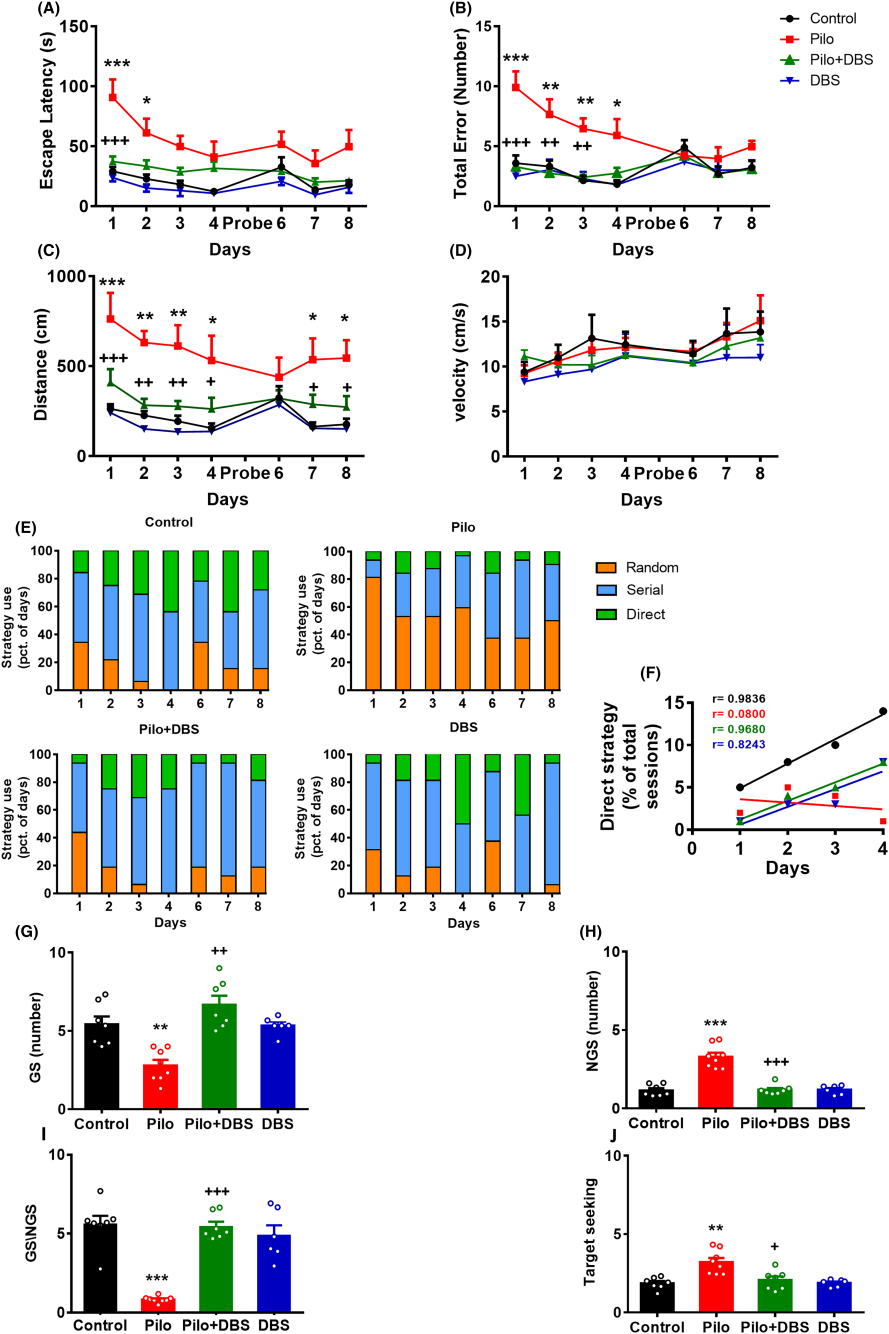
The effect of applying a closed‐loop low‐frequency DBS on spatial learning and memory by measuring different parameters of the Barnes maze test including escape latency (A), total number of errors in training days (B), distance to find the target hole (C), velocity to find the target hole (D), the changes in strategies to find the target hole (E) and the related linear regression analysis of correlation in different experimental groups (F), and the exploration frequency in the goal sector (GS) (G) and the non‐goal sector (NGS) (H), goal sector preference (GS/NGS ratio) (I), and the target‐seeking activity (J). Data are presented as means ± SEM (*n* = 7 in the control group, *n* = 6 in the DBS group, *n* = 8 in the Pilo group, and *n* = 7 in the Pilo+DBS group). *p < 0.05; **p < 0.01, and ***p < 0.001 compared to the control group. ^+^ *p* < 0.05; ^++^ *p* < 0.01, and ^+++^ *p* < 0.001 compared to the pilocarpine (Pilo) group.

A two‐way, repeated measures ANOVA followed by a Tukey's post‐hoc test revealed a significant difference between pilocarpine and control groups in learning during 4 training days. The escape latency (*F*_(3,24)_ = 11.67; *p* < 0.001), total error (*F*_(3,24)_ = 25.18; *p* < 0.001), and traveled distance (*F*_(3,24)_ = 16.75; *p* < 0.001) were greater in the pilocarpine group compared to other experimental groups, which demonstrated a significant impairment in the pilocarpine group (Figure 2). Applying DBS in the Pilo+DBS group restored the learning ability to control level. For escape latency, total number of errors, and distance traveled, there was a significant difference between Pilo+DBS and pilocarpine groups (*p* < 0.001), while no significance was observed between Pilo+DBS and control groups (Figure 2A–C). Similar results were obtained during the reversal learning phase in days 6–8: Animals in the pilocarpine group showed a significant increase in escape latency (*F*_(3,24)_ = 4.94, *p* < 0.01) and traveled distance (*F*_(3,24)_ = 5.434, *p* < 0.01) compared to the other groups, which indicated a learning impairment, and applying DBS prevented this apparent impairment (Figure 2A–C). Applying DBS in the control group had no effect on learning outcomes. There was no significant difference in the velocity among experimental groups (*F*_(3,24)_ = 0.608, *p* = 0.6 during 4 training days, and *F*_(3,24)_ = 0.481, *p* = 0.69) (Figure 2D).

For the control groups, assessing the search strategy of finding the target hole over the initial 4 days revealed that the percentage of random strategies used declined, while the direct strategy use increased during learning. In the pilocarpine group, a comparatively lower rate of direct strategy use was shown on the 4th day, while the random strategy use remained unchanged. However, in the Pilo+DBS group, the subjects' strategy was similar to the control group's strategy. During the reversal learning phase, the percentage of random strategy use decreased in the control and Pilo+DBS groups and increased in the pilocarpine group (Figure 2E,F).

In the pilocarpine group, changes in the number of goal sector (GS) explorations (*F*_(3,24)_ = 13.48, *p* < 0.001), the non‐goal sector explorations (NGS) (*F*_(3,24)_ = 34.05, *p* < 0.001), target seeking (*F*_(3,24)_ = 10.46, *p* < 0.001), and the GS/NGS ratio (as an index of GS preference) (*F*_(3,24)_ = 23.86, *p* < 0.0001) showed a significant impairment in memory on the probe day. However, applying DBS significantly restored these parameters, so that memory outcomes in the Pilo+DBS group were similar to the control group (Figure 2G–J).

### 3.3 Effect of DBS on hippocampal and mPFC oscillations

**FIGURE 3:**
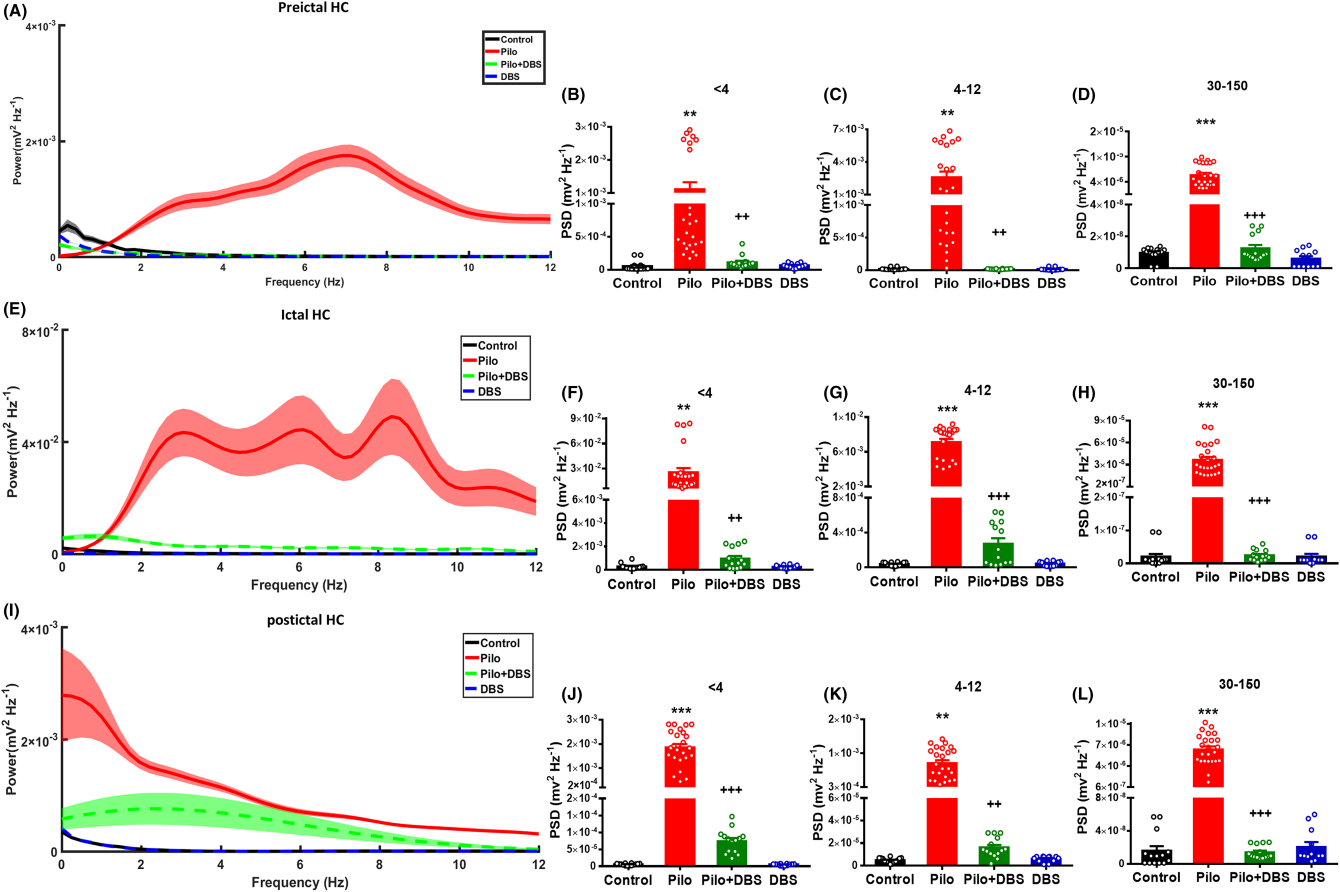
The effect of closed‐loop low‐frequency DBS on the delta, theta, and gamma power in hippocampus (HC). Part (A) shows the averaged power spectral density (PSD) of LFP recordings in pre‐ictal period. Shaded region denotes SEM. Applying DBS decreased the power spectral density of delta band (<4 Hz) (B), theta band (4–12 Hz) (C), and gamma band (30–150 Hz) (D). Section (E) shows the averaged PSD of recordings during ictal period. Shaded region denotes SEM. Applying DBS decreases the power spectral density of delta (<4 Hz) (F), theta (4–12 Hz) (G), and gamma bands (30–150 Hz) (H). Section (I) shows the averaged PSD of LFP recordings in post‐ictal period. Shaded region denotes SEM. DBS decreased the power spectral density of delta (<4 Hz) (J), theta (4–12 Hz) (K), and gamma oscillations (30–150 Hz) (L). Data are presented as means ± SEM. (Data were collected from 14 seizures/7 rats in the Pilo+DBS group and 24 seizures/8 rats in the Pilo group. In addition, 14 samples/7 rats were collected in control and 12 samples/6 rats were collected from DBS groups at similar time scales.) **p < 0.01 and ***p < 0.001 compared to the control group. ^++^ *p* < 0.01 and ^+++^ *p* < 0.001 compared to the pilocarpine (Pilo) group.

**FIGURE 4:**
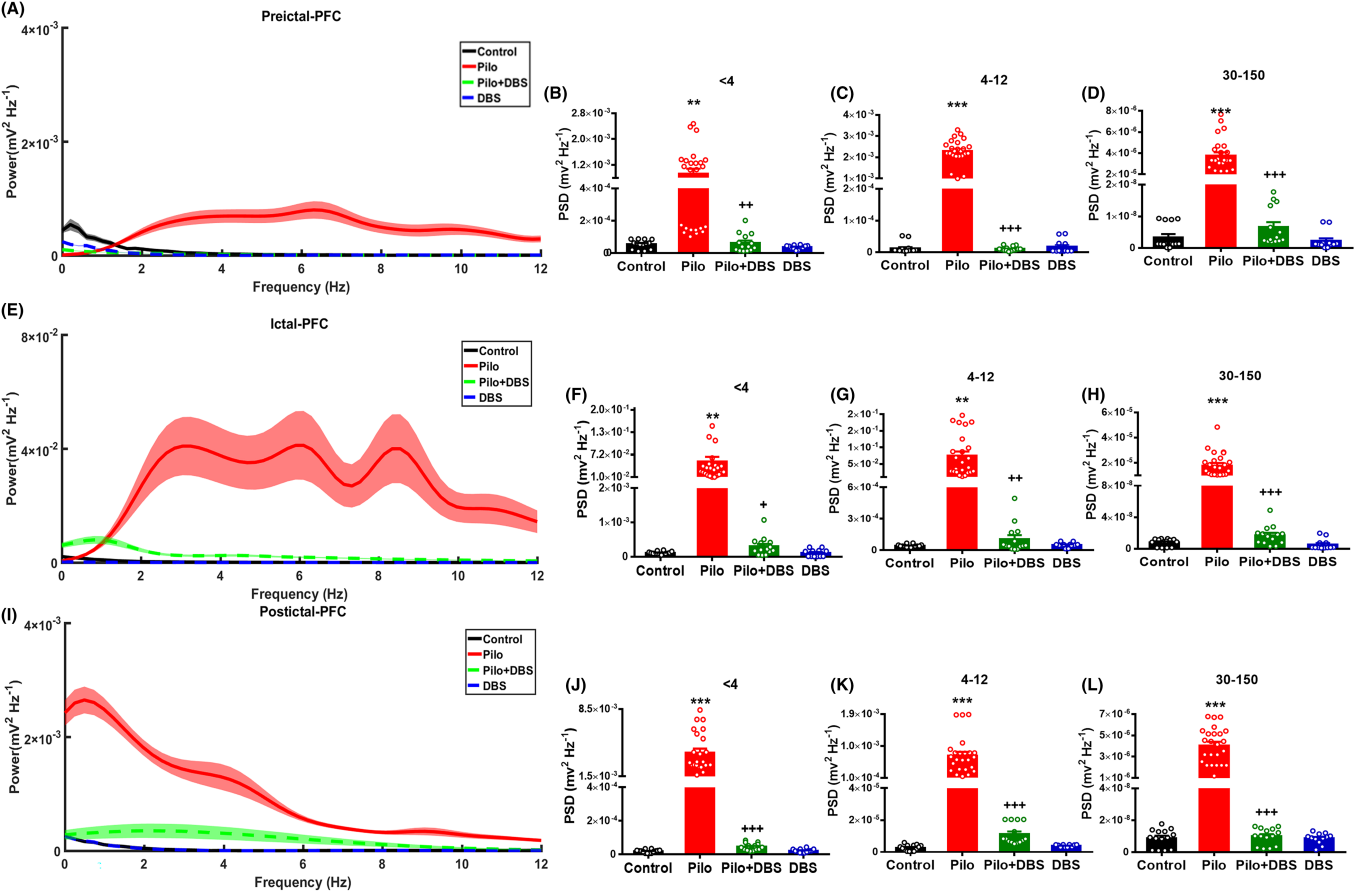
The effect of closed‐loop low‐frequency DBS on the delta, theta, and gamma power in mPFC. Part A shows the averaged power spectral density (PSD) of LFP recordings in pre‐ictal period. Shaded regions denote SEM. Applying DBS decreased the power spectral density of delta band (<4 Hz) (B), theta band (4–12 Hz) (C), and gamma band (30–150 Hz) (D). Section (E) shows the averaged PSD of recordings during ictal period. Shaded region denotes SEM. Applying DBS decreases the power spectral density of delta (<4 Hz) (F), theta (4–12 Hz) (G), and gamma bands (30–150 Hz) (H). Section (I) shows the averaged PSD of LFP recordings in the post‐ictal period. Shaded region denotes SEM. DBS decreased the power spectral density of delta (<4 Hz) (J), theta (4–12 Hz) (K), and gamma oscillations (30–150 Hz) (L). Data are presented as means ± SEM. (Data were collected from 14 seizures/7 rats in the Pilo+DBS group and 24 seizures/8 rats in the Pilo group. In addition, 14 samples/7 rats were collected in control and 12 samples/6 rats were collected from DBS groups at similar time scales.) **p < 0.01 and ***p < 0.001 compared to the control group. ^++^ *p* < 0.01 and ^+++^ *p* < 0.001 compared to the pilocarpine (Pilo) group.

The power of delta (<4 Hz), theta (4–12 Hz), and gamma (30–150 Hz) waves in the hippocampus and mPFC were evaluated at three time points: pre‐ictal (30 s before ictal onset), ictal (5 s after ictal onset), and post‐ictal (30 s after termination of ictal discharges). As Figures 3 and 4 show, in both the hippocampus and mPFC, the power of all mentioned waves was significantly higher in the pilocarpine group during pre‐ictal (delta waves: *F*_(3,60)_ = 13.34, *p* < 0.001 in hippocampus and *F*_(3,60)_ = 16.61, *p* < 0.001 in mPFC; theta waves: *F*_(3,60)_ = 13.99, *p* < 0.001 in hippocampus and *F*_(3,60)_ = 203.8, *p* < 0.001 in mPFC; and gamma waves: *F*_(3,60)_ = 42.47, *p* < 0.001 in hippocampus and *F*_(3,60)_ = 66.33, *p* < 0.001 in mPFC), ictal (delta waves: *F*_(3,60)_ = 12.94, *p* < 0.001 in hippocampus and *F*_(3,60)_ = 8.99, *p* < 0.001 in mPFC; theta waves: *F*_(3,60)_ = 191.1, *p* < 0.001 in hippocampus and *F*_(3,60)_ = 18.6, *p* < 0.001 in mPFC; and gamma waves: *F*_(3,60)_ = 42.55, *p* < 0.001 in hippocampus and *F*_(3,60)_ = 38.97, *p* < 0.001 in mPFC), and post‐ictal periods (delta waves: *F*_(3,60)_ = 78.67, *p* < 0.001 in hippocampus and *F*_(3,60)_ = 51.56, *p* < 0.001 in mPFC; theta waves: *F*_(3,60)_ = 71.93, *p* < 0.001 in hippocampus and *F*_(3,60)_ = 23.13, *p* < 0.001 in mPFC; and gamma waves: *F*_(3,60)_ = 99.17, *p* < 0.001 in hippocampus and *F*_(3,60)_ = 7989, *p* < 0.001 in mPFC). However, in the Pilo+DBS group, the power of all oscillations was significantly lower than in the pilocarpine group, and there were no differences between Pilo+DBS and control groups. Applying DBS alone had no effect on power. The same changes were observed in gamma sub‐bands (slow, middle, and fast gamma) (see Figures S2 and S3).

### 3.4 Effect of DBS on coherence connectivity

**FIGURE 5:**
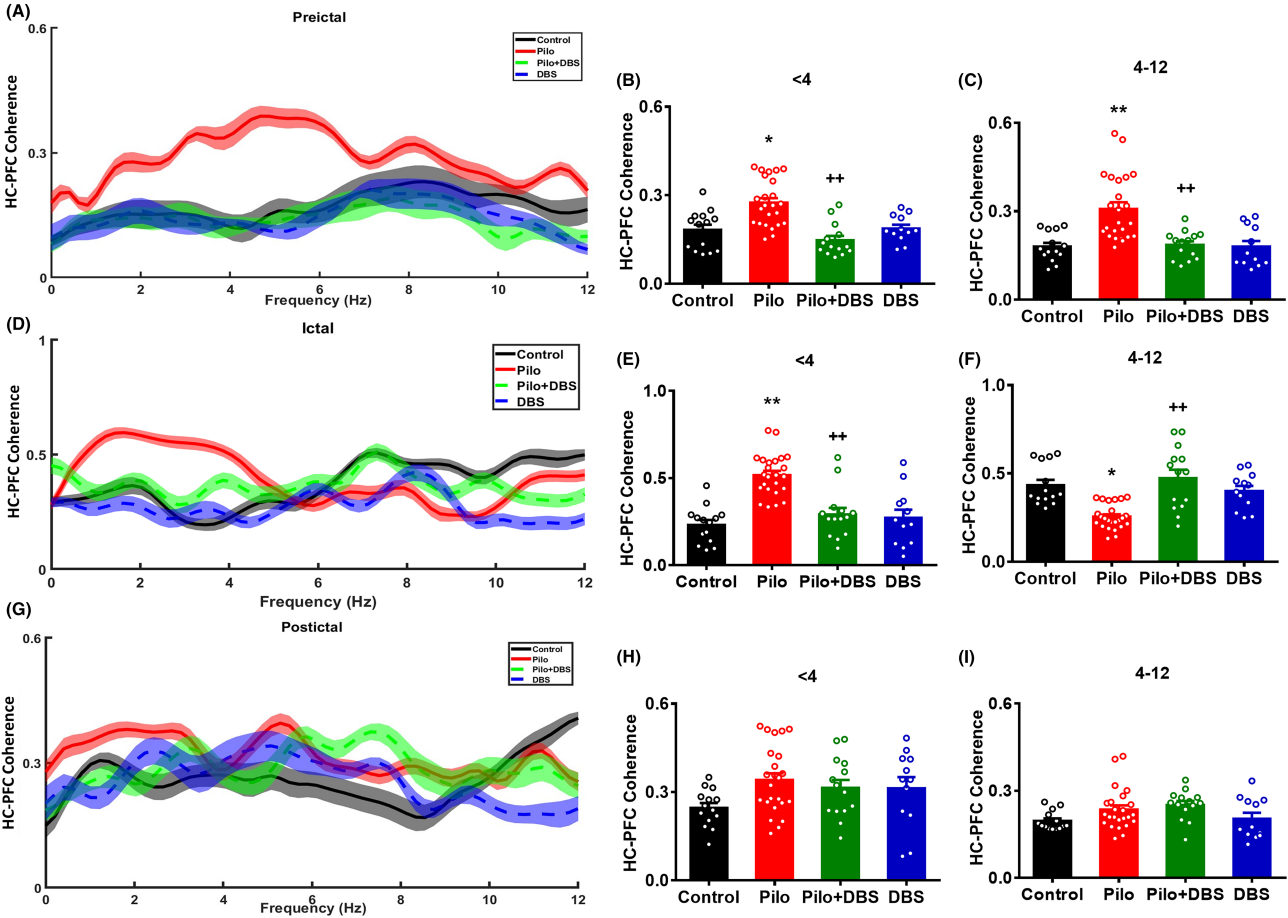
The effect of closed‐loop low‐frequency DBS on the coherence between hippocampus (HC) and mPFC at delta and theta frequencies. (A) Coherence spectra between HC and mPFC in delta and theta frequencies (<12 Hz) in pre‐ictal period. Shaded area indicates SEM. Bar diagrams show the coherence between HC‐mPFC in delta (B) and theta frequencies (C). Section (D) shows the coherence spectra between HC and mPFC in delta and theta frequencies (<12 Hz) during ictal period. Shaded area indicates SEM. Bar diagrams show coherence between HC and mPFC in delta (E) and theta frequencies (F). Section (G) shows the coherence spectra between HC‐mPFC in delta and theta frequencies (<12 Hz) in the post‐ictal period. Shaded area indicates SEM. Bar diagrams show coherence between HC and mPFC in delta (H) and theta oscillations (I). Data are presented as means ± SEM. (Data were collected from 14 seizures/7 rats in the Pilo+DBS group and 24 seizures/8 rats in the Pilo group. In addition, 14 samples/7 rats were collected in control and 12 samples/6 rats were collected from DBS groups at similar time scales.) *p < 0.05 and **p < 0.01 compared to the control group. ^++^ *p* < 0.01 compared to the pilocarpine (Pilo) group.

In the next step, we analyzed the inter‐regional LFP interactions by calculating the coherence across the frequency spectrum, which measures the stability of the difference between two signals at the same frequency. A one‐way ANOVA showed significant increase in delta and theta bands coherence between hippocampus and mPFC compared to the control group during pre‐ictal period (*F*_(3,60)_ = 12.92, *p* < 0.001 and *F*_(3,60)_ = 12.13, *p* < 0.001 for delta and theta bands, respectively) (Figure 5A–C). Applying DBS in Pilo+DBS group reduced the coherence of delta and theta bands, and Tukey's post‐hoc test showed a significant difference between Pilo+DBS and pilocarpine groups (*p* < 0.01) (Figure 5A–C). A one‐way ANOVA showed significant difference in the coherence of delta (*F*_(3,60)_ = 19.04, *p* < 0.001) and theta (*F*_(3,60)_ = 13.22, *p* < 0.001) bands between hippocampus and mPFC among experimental groups. During ictal period, the coherence of the delta band in the pilocarpine group was higher, but of theta band was lower than the control group (*p* < 0.01). Applying DBS in the Pilo+DBS group prevented the changes in coherence compared with the pilocarpine group (*p* < 0.01) (Figure 5D–F). The coherence between the hippocampus and mPFC in the delta and theta bands in the post‐ictal period had no significant difference among experimental groups (*F*_(3,60)_ = 2.35, *p* = 0.081 and *F*_(3,60)_ = 2.66, *p* = 0.06 for delta and theta bands, respectively) (Figure 5G–I).

### 3.5 Effect of DBS on phase–amplitude coupling between hippocampus and mPFC

**FIGURE 6:**
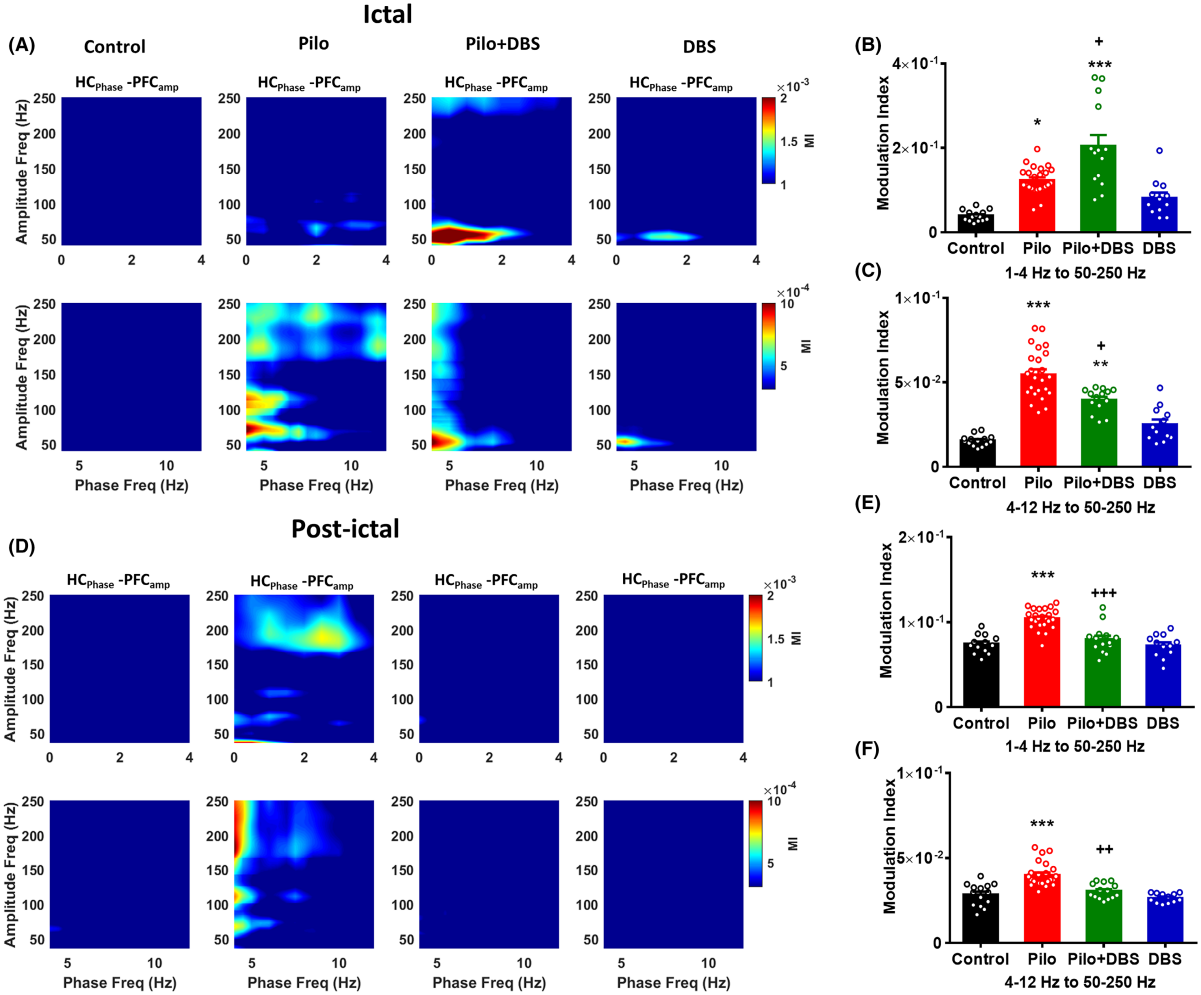
The effect of applying a closed‐loop low‐frequency DBS on the cross‐frequency coupling of hippocampus and mPFC areas. Part (A) shows the representative comodulogram of modulation index computed for hippocampal delta and theta phases (1–12 Hz) and mPFC gamma amplitude (50–250 Hz) during ictal phase. The bar graphs represent mean values of modulation index for hippocampal delta phase to mPFC gamma amplitude (B) and hippocampal theta phase to mPFC gamma amplitude (C). Part (D) shows the representative comodulogram of modulation index computed for hippocampal delta and theta phases (1–12 Hz) and mPFC gamma amplitude (50–250 Hz) during post‐ictal phase. The bar graphs represent mean values of modulation index for hippocampal delta phase to mPFC gamma amplitude (E) and hippocampal theta phase to mPFC gamma amplitude (F). Data are presented as means ± SEM. (Data were collected from 14 seizures/7 rats in the Pilo+DBS group and 24 seizures/8 rats in the Pilo group. In addition, 14 samples/7 rats were collected in control and 12 samples/6 rats were collected from DBS groups at similar time scales.) *p < 0.05; **p < 0.01, and ***p < 0.001 compared to the control group. ^+^ *p* < 0.05; ^++^ *p* < 0.01, and ^+++^ *p* < 0.001 compared to the pilocarpine (Pilo) group.

In the next step, we examined LFP interactions among oscillations of inter‐regional different frequencies. These are commonly called cross‐frequency coupling (CFC) or co‐modulation.^32^, ^33^ Here, we focused on phase–amplitude coupling – a relationship that is hypothesized as a mechanism for functional communication between local and global circuits.^34^ We calculated the inter‐regional CFC by analyzing the hippocampal phase and the mPFC amplitude. In the pre‐ictal period, there was no significant difference in CFC between the pilocarpine and the control group (Figure 6A–C). However, during the ictal phase, a one‐way ANOVA showed significant difference among experimental groups in coupling between delta and gamma (1–4 Hz to 50–250 Hz) (*F*_(3,60)_ = 23.59, *p* < 0.001; Figure 6A,B) and between theta and gamma (4–12 Hz to 50–250 Hz) (*F*_(3,60)_ = 46.87, *p* < 0.001; Figure 6A,C). A Tukey's post hoc test showed a significant increase in coupling between delta and gamma (*p* < 0.05) and between theta and gamma (*p* < 0.001) in the pilocarpine compared to the control group. Applying DBS in the Pilo+DBS group significantly increased the delta–gamma coupling (*p* < 0.05) and decreased the theta–gamma coupling (*p* < 0.05) compared to the pilocarpine group (Figure 6A–C).

During post‐ictal period, there was also significant difference among experimental groups in coupling between delta and gamma (*F*_(3,60)_ = 24.51, *p* < 0.001; Figure 6D,E) and between theta and gamma (*F*_(3,60)_ = 20.43, *p* < 0.001; Figure 6D,F). Similar to ictal period, Tukey's post hoc test showed a significant increase in coupling between delta and gamma (*p* < 0.001) and between theta and gamma (*p* < 0.001) in the pilocarpine compared to the control group. Applying DBS in the Pilo+DBS group significantly decreased the delta–gamma coupling (*p* < 0.001) and the theta–gamma coupling (*p* < 0.01) compared to the pilocarpine group (Figure 6D–F).

**FIGURE 7:**
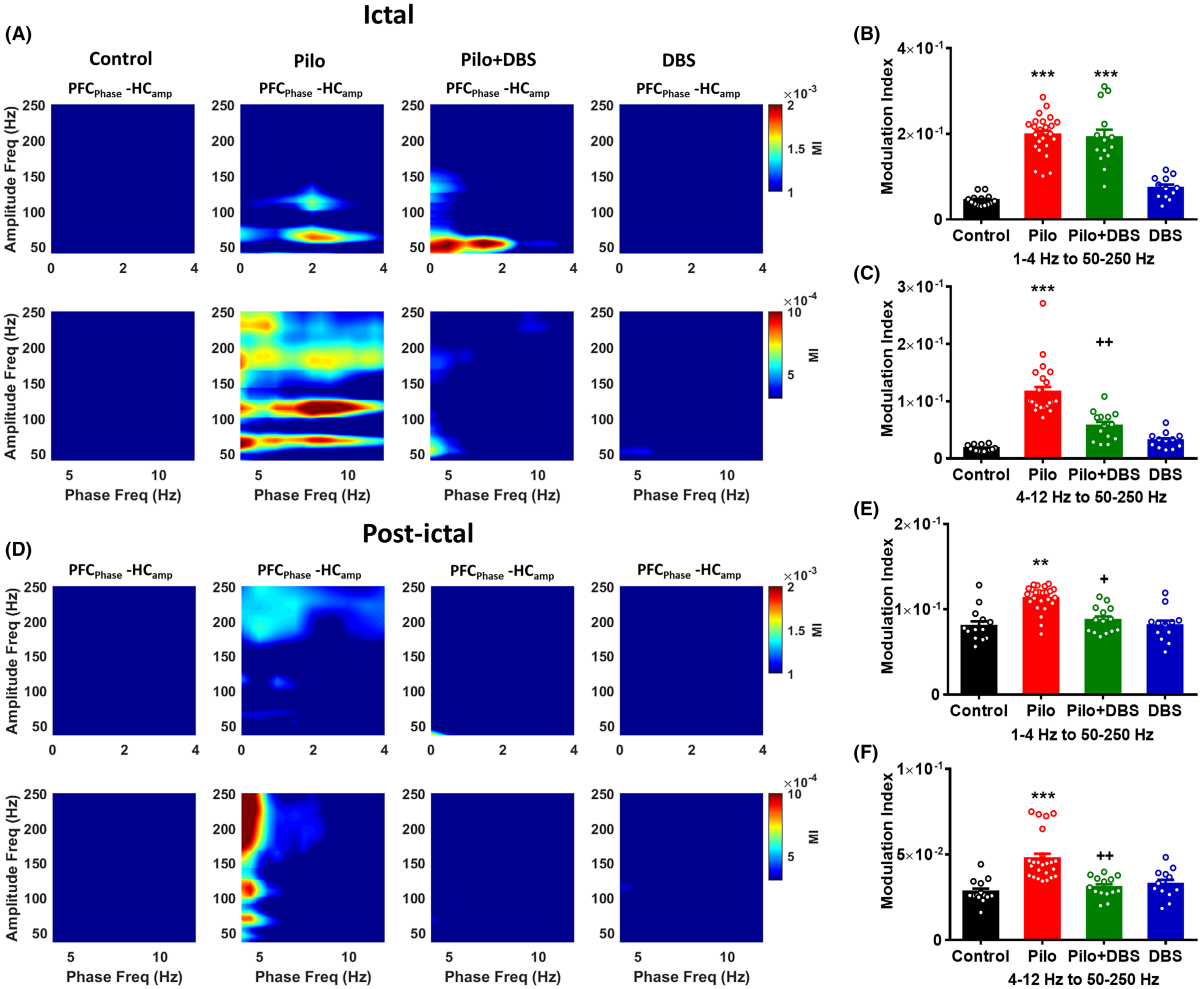
The effect of applying a closed‐loop low‐frequency DBS on the cross‐frequency coupling of mPFC and hippocampal areas. Part (A) shows the representative comodulogram of modulation index computed for mPFC delta and theta phases (1–12 Hz) and hippocampal gamma amplitude (50–250 Hz) during ictal phase. The bar graphs represent mean values of modulation index for mPFC delta phase to hippocampal gamma amplitude (B) and mPFC theta phase to hippocampal gamma amplitude (C). Part (D) shows the representative comodulogram of modulation index computed for mPFC delta and theta phases (1–12 Hz) and hippocampal gamma amplitude (50–250 Hz) during post‐ictal phase. The bar graphs represent mean values of modulation index for mPFC delta phase to hippocampal gamma amplitude (E) and mPFC theta phase to hippocampal gamma amplitude (F). Data are presented as means ± SEM. (Data were collected from 14 seizures/7 rats in the Pilo+DBS group and 24 seizures/8 rats in the Pilo group. In addition, 14 samples/7 rats were collected in control and 12 samples/6 rats were collected from DBS groups at similar time scales.) **p < 0.01, and ***p < 0.001 compared to the control group. ^+^ *p* < 0.05, and ^++^ *p* < 0.01 compared to the pilocarpine (Pilo) group.

Similar data were obtained when we calculated the inter‐regional CFC by analyzing the mPFC phase and the hippocampal amplitude (Figure 7). During the ictal period, a one‐way ANOVA showed significant difference among experimental groups in coupling between delta and gamma (*F*_(3,60)_ = 47.95, *p* < 0.001; Figure 7A,B) and between theta and gamma (*F*_(3,60)_ = 40.03, *p* < 0.001; Figure 7A,C). A Tukey's post‐hoc test showed a significant increase in coupling between delta and gamma and between theta and gamma (*p* < 0.001) in the pilocarpine compared to the control group. Applying DBS in the Pilo+DBS group only decreased the theta–gamma coupling (*p* < 0.01) compared to the pilocarpine group (Figure 7A–C).

In the post‐ictal period, a one‐way ANOVA showed significant difference among experimental groups in coupling between delta and gamma (*F*_(3,60)_ = 16.03, *p* < 0.001; Figure 7D,E) and between theta and gamma (*F*_(3,60)_ = 15.07, *p* < 0.001; Figure 7D,F). A Tukey's post hoc test showed a significant increase in coupling between delta and gamma (*p* < 0.01) and between theta and gamma (*p* < 0.001) in the pilocarpine compared to the control group. Applying DBS in the Pilo+DBS group decreased the delta–gamma coupling (*p* < 0.05) and the theta–gamma coupling (*p* < 0.01) compared to the pilocarpine group (Figure 7D–F).

Similar results were obtained by measuring the CFC in the hippocampal delta and theta phases and its gamma amplitude (see Figure S6) and in the mPFC delta and theta phases and its gamma amplitude (see Figure S7).

## 4 DISCUSSION

Results showed that closed‐loop low‐frequency DBS had anticonvulsant effects that were accompanied by returning the neuronal oscillations and connectivity toward a non‐seizure state and restoring the learning and memory performance. We used a closed‐loop stimulation to mimic the RNS (responsive brain stimulation) system, which is an FDA‐approved treatment for adults with drug‐resistant focal epilepsy.^35^

In the pilocarpine model of epilepsy, the primary origin of seizures is the ventral hippocampus and ventral subiculum.^36^ Applying closed‐loop low‐frequency (1 Hz) DBS in the hippocampus reduced the number and duration of spontaneous epileptic seizures. These results were similar to previous reports that showed applying a closed‐loop DBS at 5 Hz^37^ and 20 Hz^38^ reduced seizure severity in epileptic subjects. Although high‐frequency DBS has been approved by the FDA for the treatment of epilepsy in human patients,^39^ our data confirmed that low‐frequency DBS is also very effective. Considering the lower potential for brain damage and lower battery consumption following low‐frequency (compared to high frequency) DBS, closed‐loop, low‐frequency DBS may be among the best choices in epilepsy treatment.

DBS observed anticonvulsant actions were accompanied by changes in the hippocampal and mPFC connectivity. Abnormal communication in the prefrontal–hippocampal pathway may be involved in the occurrence of seizures^4^, ^5^, ^6^ and has been suggested as a marker for TLE.^40^, ^41^ In addition, the occurrence of epileptiform discharges may disconnect the hippocampal–cortical coupling, thereby impairing the cognitive domains, such as learning and memory, in epilepsy patients.^42^

The connection between the hippocampus and prefrontal cortex is essential for memory function.^43^ Our results showed learning and spatial memory impairment following pilocarpine‐induced epilepsy. This impairment was reduced following DBS application. Consistent with the present results, we previously showed that open‐loop, low‐frequency DBS restores learning and memory in kindled rats.^44^ Similar results were obtained by applying high‐frequency DBS in a closed‐loop system in patients with epilepsy: following DBS, patients verbal memory tasks performance improved.^16^

Our results also showed that DBS improved the performance on reversal learning. Reversal learning is impaired in absence of epilepsy patients.^45^ The PFC has an important role in reversal learning processes, and damage to the PFC impairs reversal learning.^46^ Low‐frequency DBS may restore reversal learning by impacting neuronal oscillations in the mPFC and returning these oscillations toward normal values. This may be an important aspect of low‐frequency DBS therapeutic action.

Applying DBS in epileptic animals restored the power of delta, theta, and gamma bands to normal state. Delta bands may be an indicator of seizures^47^ and there is a direct relationship between the delta band and the occurrence of seizures.^48^ Previous studies also showed that theta band power is particularly pronounced at the onset of seizures^49^ and during the preictal period.^40^ In addition, gamma oscillations precede the onset of ictal discharges in humans.^50^ Therefore, restoring the power of delta, theta, and gamma waves by DBS may be involved in its anticonvulsant effect. In addition, our data showed an increase in the ratio of theta coherence and power in the post‐ictal to the pre‐ictal period (see Figures S4 and S5). This may be a cause for the post‐DBS improvement in learning and memory, when we consider the role of theta waves in cognitive functions.

DBS application reduced the hypersynchrony (i.e., the increase in coherence) in theta bands between hippocampus and mPFC in animals with spontaneous seizures. Synchrony between the hippocampus and mPFC in epilepsy may result in learning and memory impairment.^42^, ^51^ We hypothesized that an abnormal decrease or increase in the coherence may disrupt memory processes. Our hypothesis is based on previous reports showing that abnormal, seizure‐induced synaptic potentiation impairs the new synaptic potentiation and results in memory impairment.^29^, ^44^ In this regard, it has been suggested that an abnormal plasticity may occur in mPFC communications following the strong coupling of hippocampal theta and mPFC gamma bands.^52^

Although there was no significant difference in coherence of delta and theta bands during post‐ictal period, calculating the ratio between post‐ictal and pre‐ictal has shown an increase in delta and theta coherence after DBS application.

Our results showed a significant increase in theta–gamma phase–frequency coupling in the hippocampus and mPFC in pilocarpine seizure‐induced animals during ictal and post‐ictal periods. Low‐frequency DBS reduced this effect. In healthy animals, the coupling between theta in the hippocampus and gamma in the mPFC may influence plasticity in the prefrontal cortex,^53^ which can likely improve learning and spatial memory. However, the abnormal theta–gamma coupling may involve in some neurological diseases.^54^ Therefore, the restoring effect of DBS on this parameter may also involve in its therapeutic action.

Post‐ictally, we observed that applying DBS reduced the coupling between delta and theta with gamma and higher‐frequency oscillations. The relationship between changes in theta phase and slow gamma has been shown in spatial memory disorders in epileptic rats.^55^ Therefore, the reduction in phase–amplitude coupling may be a mechanism by which DBS may exert its antiepileptic effect.^56^

## 5 CONCLUSION

Taken together, the present study shows that applying closed‐loop, low‐frequency DBS had anticonvulsant effects in rats with pilocarpine‐induced epilepsy, and reduced the seizure‐induced impairments in learning and memory. In addition, the hippocampus–mPFC functional connectivity was restored to pre‐seizure levels after DBS was applied to rats with pilocarpine‐induced seizures. Therefore, more studies are needed to shed light on the effects of DBS on brain rhythms and the functional connectivity between areas implicated in epilepsy and other brain areas.

## AUTHOR CONTRIBUTIONS

All authors contributed to the study conception and design. Conceptualization: Javad Mirnajafi‐Zadeh, Amir Shojaei, and Mohammad Reza Raoufy; Methodology: Meysam Zare, Mahmoud Rezaei, Milad Nazari, Nastaran Kosarmadar, and Mona Faraz; Data analysis: Meysam Zare; Writing—original draft preparation: Meysam Zare; Writing—review and editing: Javad Mirnajafi‐Zadeh and Victoria Barkely; Funding acquisition: Javad Mirnajafi‐Zadeh; and Supervision: Javad Mirnajafi‐Zadeh. All authors read and approved the final manuscript.

## CONFLICT OF INTEREST STATEMENT

The authors declare no conflicts of interest.

## Supporting information


Figures S1–S7

## Data Availability

Data will be available under a request.

## References

[1] Thijs RD , Surges R , O'Brien TJ , Sander JW . Epilepsy in adults. Lancet. 2019;393(10172):689‐701. 30686584 10.1016/S0140-6736(18)32596-0

[2] Jiruska P , De Curtis M , Jefferys JG , Schevon CA , Schiff SJ , Schindler K . Synchronization and desynchronization in epilepsy: controversies and hypotheses. J Physiol. 2013;591(4):787‐797. 23184516 10.1113/jphysiol.2012.239590 PMC3591697

[3] Jefferys JG , Jiruska P , de Curtis M , Avoli M . Limbic network synchronization and temporal lobe epilepsy. In: Noebels JL , Avoli M , Rogawski MA , Olsen RW , Delgado‐Escueta AV , eds. Jasper's Basic Mechanisms of the Epilepsies [Internet]. 4th ed. National Center for Biotechnology Information (US); 2012. 22787650

[4] Kemmotsu N , Kucukboyaci NE , Leyden KM , et al. Frontolimbic brain networks predict depressive symptoms in temporal lobe epilepsy. Epilepsy Res. 2014;108(9):1554‐1563. 25223729 10.1016/j.eplepsyres.2014.08.018 PMC4194230

[5] Kleen JK , Wu EX , Holmes GL , Scott RC , Lenck‐Santini P‐P . Enhanced oscillatory activity in the hippocampal–prefrontal network is related to short‐term memory function after early‐life seizures. J Neurosci. 2011;31(43):15397‐15406. 22031886 10.1523/JNEUROSCI.2196-11.2011 PMC3224083

[6] Holmes GL . Cognitive impairment in epilepsy: the role of network abnormalities. Epileptic Disord. 2015;17(2):101‐116. 25905906 10.1684/epd.2015.0739 PMC5410366

[7] O'Neill P‐K , Gordon JA , Sigurdsson T . Theta oscillations in the medial prefrontal cortex are modulated by spatial working memory and synchronize with the hippocampus through its ventral subregion. J Neurosci. 2013;33(35):14211‐14224. 23986255 10.1523/JNEUROSCI.2378-13.2013 PMC3756763

[8] Eichenbaum H . Prefrontal–hippocampal interactions in episodic memory. Nat Rev Neurosci. 2017;18(9):547‐558. 28655882 10.1038/nrn.2017.74

[9] Manns JR , Hopkins RO , Reed JM , Kitchener EG , Squire LR . Recognition memory and the human hippocampus. Neuron. 2003;37(1):171‐180. 12526782 10.1016/s0896-6273(02)01147-9

[10] D'Hooge R , De Deyn PP . Applications of the Morris water maze in the study of learning and memory. Brain Res Rev. 2001;36(1):60‐90. 11516773 10.1016/s0165-0173(01)00067-4

[11] Lopes MW , Lopes SC , Santos DB , et al. Time course evaluation of behavioral impairments in the pilocarpine model of epilepsy. Epilepsy Behav. 2016;55:92‐100. 26773677 10.1016/j.yebeh.2015.12.001

[12] Fuster JM . Cognitive functions of the prefrontal cortex. In: Stuss DT , Knight RT , eds. Principles of Frontal Lobe Function. Oxford University Press; 2013:11‐22.

[13] Bizon JL , Foster TC , Alexander GE , Glisky EL . Characterizing cognitive aging of working memory and executive function in animal models. Front Aging Neurosci. 2012;4:19. 22988438 10.3389/fnagi.2012.00019 PMC3439637

[14] Guerreiro CA , Jones‐Gotman M , Andermann F , Bastos A , Cendes F . Severe amnesia in epilepsy: causes, anatomopsychological considerations, and treatment. Epilepsy Behav. 2001;2(3):224‐246. 12609367 10.1006/ebeh.2001.0167

[15] Li MC , Cook MJ . Deep brain stimulation for drug‐resistant epilepsy. Epilepsia. 2018;59(2):273‐290. 29218702 10.1111/epi.13964

[16] Ezzyat Y , Wanda PA , Levy DF , et al. Closed‐loop stimulation of temporal cortex rescues functional networks and improves memory. Nat Commun. 2018;9(1):365. 29410414 10.1038/s41467-017-02753-0 PMC5802791

[17] Velíšek L , Velíšková J , Moshé SL . Electrical stimulation of substantia nigra pars reticulata is anticonvulsant in adult and young male rats. Exp Neurol. 2002;173(1):145‐152. 11771947 10.1006/exnr.2001.7830

[18] Tykocki T , Mandat T , Kornakiewicz A , Koziara H , Nauman P . State of the art paper deep brain stimulation for refractory epilepsy. Arch Med Sci. 2012;8(5):805‐816. 23185188 10.5114/aoms.2012.31135 PMC3506228

[19] Boon P , Vonck K , De Herdt V , et al. Deep brain stimulation in patients with refractory temporal lobe epilepsy. Epilepsia. 2007;48(8):1551‐1560. 17726798 10.1111/j.1528-1167.2007.01005.x

[20] Peltola J , Colon AJ , Pimentel J , et al. Deep brain stimulation of the anterior nucleus of the thalamus in drug‐resistant epilepsy in the MORE multicenter patient registry. Neurology. 2023;100(18):e1852‐e1865. 36927882 10.1212/WNL.0000000000206887 PMC10159763

[21] Ghotbedin Z , Janahmadi M , Mirnajafi‐Zadeh J , Behzadi G , Semnanian S . Electrical low frequency stimulation of the kindling site preserves the electrophysiological properties of the rat hippocampal CA1 pyramidal neurons from the destructive effects of amygdala kindling: the basis for a possible promising epilepsy therapy. Brain Stimul. 2013;6(4):515‐523. 23228730 10.1016/j.brs.2012.11.001

[22] Kile KB , Tian N , Durand DM . Low frequency stimulation decreases seizure activity in a mutation model of epilepsy. Epilepsia. 2010;51(9):1745‐1753. 20659150 10.1111/j.1528-1167.2010.02679.x PMC3569726

[23] Velíšek L , Velíšková J , Stanton PK . Low‐frequency stimulation of the kindling focus delays basolateral amygdala kindling in immature rats. Neurosci Lett. 2002;326(1):61‐63. 12052538 10.1016/s0304-3940(02)00294-x

[24] Esmaeilpour K , Sheibani V , Shabani M , Mirnajafi‐Zadeh J . Effect of low frequency electrical stimulation on seizure‐induced short‐and long‐term impairments in learning and memory in rats. Physiol Behav. 2017;168:112‐121. 27825910 10.1016/j.physbeh.2016.11.001

[25] Ghafouri S , Fathollahi Y , Javan M , Shojaei A , Asgari A , Mirnajafi‐Zadeh J . Effect of low frequency stimulation on impaired spontaneous alternation behavior of kindled rats in Y‐maze test. Epilepsy Res. 2016;126:37‐44. 27423017 10.1016/j.eplepsyres.2016.06.010

[26] Ghasemi Z , Naderi N , Shojaei A , et al. The inhibitory effect of different patterns of low frequency stimulation on neuronal firing following epileptiform activity in rat hippocampal slices. Brain Res. 2019;1706:184‐195. 30419223 10.1016/j.brainres.2018.11.012

[27] Racine RJ . Modification of seizure activity by electrical stimulation: II. Motor seizure. Electroencephalogr Clin Neurophysiol. 1972;32(3):281‐294. 4110397 10.1016/0013-4694(72)90177-0

[28] Paxinos G , Watson C . The Rat Brain in Stereotaxic Coordinates. 6th ed. Academic Press; 2006.

[29] Faraz M , Kosarmadar N , Rezaei M , et al. Deep brain stimulation effects on learning, memory and glutamate and GABAA receptor subunit gene expression in kindled rats. Acta Neurobiol Exp. 2021;81(1):43‐57. 10.21307/ane-2021-006 33949168

[30] Zare M , Nazari M , Shojaei A , Raoufy MR , Mirnajafi‐Zadeh J . Online analysis of local field potentials for seizure detection in freely moving rats. Iran J Basic Med Sci. 2020;23(2):173‐177. 32405359 10.22038/IJBMS.2019.38722.9183 PMC7211346

[31] Tort AB , Komorowski R , Eichenbaum H , Kopell N . Measuring phase‐amplitude coupling between neuronal oscillations of different frequencies. J Neurophysiol. 2010;104(2):1195‐1210. 20463205 10.1152/jn.00106.2010 PMC2941206

[32] Jensen O , Colgin LL . Cross‐frequency coupling between neuronal oscillations. Trends Cogn Sci. 2007;11(7):267‐269. 17548233 10.1016/j.tics.2007.05.003

[33] Scheffer‐Teixeira R , Tort ABL . Theta‐gamma cross‐frequency analyses (hippocampus). In: Jaeger D , Jung R , eds. Encyclopedia of Computational Neuroscience. Springer; 2018:1‐15.

[34] Canolty RT , Knight RT . The functional role of cross‐frequency coupling. Trends Cogn Sci. 2010;14(11):506‐515. 20932795 10.1016/j.tics.2010.09.001 PMC3359652

[35] Jarosiewicz B , Morrell M . The RNS system: brain‐responsive neurostimulation for the treatment of epilepsy. Expert Rev Med Devices. 2021;18(2):129‐138. 10.1080/17434440.2019.1683445 32936673

[36] Toyoda I , Bower MR , Leyva F , Buckmaster PS . Early activation of ventral hippocampus and subiculum during spontaneous seizures in a rat model of temporal lobe epilepsy. J Neurosci. 2013;33(27):11100‐11115. 23825415 10.1523/JNEUROSCI.0472-13.2013 PMC3718374

[37] Salam MT , Velazquez JLP , Genov R . Seizure suppression efficacy of closed‐loop versus open‐loop deep brain stimulation in a rodent model of epilepsy. IEEE Trans Neural Syst Rehabil Eng. 2015;24(6):710‐719. 26571534 10.1109/TNSRE.2015.2498973

[38] Takeuchi Y , Harangozó M , Pedraza L , et al. Closed‐loop stimulation of the medial septum terminates epileptic seizures. Brain. 2021;144(3):885‐908. 33501929 10.1093/brain/awaa450

[39] Fisher R , Salanova V , Witt T , et al. Electrical stimulation of the anterior nucleus of thalamus for treatment of refractory epilepsy. Epilepsia. 2010;51(5):899‐908. 20331461 10.1111/j.1528-1167.2010.02536.x

[40] Broggini ACS , Esteves IM , Romcy‐Pereira RN , Leite JP , Leao RN . Pre‐ictal increase in theta synchrony between the hippocampus and prefrontal cortex in a rat model of temporal lobe epilepsy. Exp Neurol. 2016;279:232‐242. 26953232 10.1016/j.expneurol.2016.03.007

[41] Kitchigina VF . Alterations of coherent theta and gamma network oscillations as an early biomarker of temporal lobe epilepsy and Alzheimer's disease. Front Integr Neurosci. 2018;12:36. 30210311 10.3389/fnint.2018.00036 PMC6119809

[42] Naik AA , Brodovskaya A , Subedi S , Akram A , Kapur J . Extrahippocampal seizure and memory circuits overlap. eNeuro. 2022;9(4):ENEURO.0179‐22.2022. 10.1523/ENEURO.0179-22.2022 PMC9319425 35853724

[43] Remondes M , Schuman EM . Role for a cortical input to hippocampal area CA1 in the consolidation of a long‐term memory. Nature. 2004;431(7009):699‐703. 15470431 10.1038/nature02965

[44] Sadeghian A , Salari Z , Azizi H , et al. The role of dopamine D2‐like receptors in a “depotentiation‐like effect” of deep brain stimulation in kindled rats. Brain Res. 2020;1738:146820. 32251663 10.1016/j.brainres.2020.146820

[45] Roebuck AJ , An L , Marks WN , Sun N , Snutch TP , Howland JG . Cognitive impairments in touchscreen‐based visual discrimination and reversal learning in genetic absence epilepsy rats from Strasbourg. Neuroscience. 2020;430:105‐112. 32017953 10.1016/j.neuroscience.2020.01.028

[46] Bartolo R , Averbeck BB . Prefrontal cortex predicts state switches during reversal learning. Neuron. 2020;106(6):1044‐1054.e4. 32315603 10.1016/j.neuron.2020.03.024 PMC7422923

[47] Khamis H , Mohamed A , Simpson S . Seizure state detection of temporal lobe seizures by autoregressive spectral analysis of scalp EEG. Clin Neurophysiol. 2009;120(8):1479‐1488. 19564130 10.1016/j.clinph.2009.05.016

[48] Tao JX , Chen XJ , Baldwin M , et al. Interictal regional delta slowing is an EEG marker of epileptic network in temporal lobe epilepsy. Epilepsia. 2011;52(3):467‐476. 21204828 10.1111/j.1528-1167.2010.02918.x

[49] Kitchigina VF , Butuzova MV . Theta activity of septal neurons during different epileptic phases: the same frequency but different significance? Exp Neurol. 2009;216(2):449‐458. 19168062 10.1016/j.expneurol.2009.01.001

[50] Alvarado‐Rojas C , Valderrama M , Fouad‐Ahmed A , et al. Slow modulations of high‐frequency activity (40–140 Hz) discriminate preictal changes in human focal epilepsy. Sci Rep. 2014;4(1):4545. 24686330 10.1038/srep04545 PMC3971396

[51] Sigurdsson T , Duvarci S . Hippocampal‐prefrontal interactions in cognition, behavior and psychiatric disease. Front Syst Neurosci. 2016;9:190. 26858612 10.3389/fnsys.2015.00190 PMC4727104

[52] Zheng C , Zhang T . Alteration of phase–phase coupling between theta and gamma rhythms in a depression‐model of rats. Cogn Neurodyn. 2013;7:167‐172. 24427199 10.1007/s11571-012-9225-x PMC3595434

[53] Zheng C , Zhang T . Synaptic plasticity‐related neural oscillations on hippocampus–prefrontal cortex pathway in depression. Neuroscience. 2015;292:170‐180. 25684752 10.1016/j.neuroscience.2015.01.071

[54] Zhang X , Zhong W , Brankačk J , et al. Impaired theta‐gamma coupling in APP‐deficient mice. Sci Rep. 2016;6(1):1‐10. 26905287 10.1038/srep21948 PMC4764939

[55] Lopez‐Pigozzi D , Laurent F , Brotons‐Mas JR , et al. Altered oscillatory dynamics of CA1 parvalbumin basket cells during theta–gamma rhythmopathies of temporal lobe epilepsy. eNeuro. 2016;3(6):ENEURO.0284‐16.2016. 10.1523/ENEURO.0284-16.2016 PMC5114702 27896315

[56] Mihály I , Orbán‐Kis K , Gáll Z , Berki Á‐J , Bod R‐B , Szilágyi T . Amygdala low‐frequency stimulation reduces pathological phase‐amplitude coupling in the pilocarpine model of epilepsy. Brain Sci. 2020;10(11):856. 33202818 10.3390/brainsci10110856 PMC7696538

