# Supplementary material for: Effect of the closed‐loop hippocampal low‐frequency stimulation on seizure severity, learning, and memory in pilocarpine epilepsy rat model
Source: CNS Neurosci Ther. 2024 Mar 4;30(3):e14656. doi: 10.1111/cns.14656 (PMC10912795; doi:10.1111/cns.14656)
Supplement: Supplementary file 1 — Figures S1–S7 [file CNS-30-e14656-s001.pdf]

Effect of the closed-loop hippocampal stimulation on seizures severity, learning and memory in the pilocarpine-induced epilepsy rat model

Supplementary figures

Supplementary Fig. 1

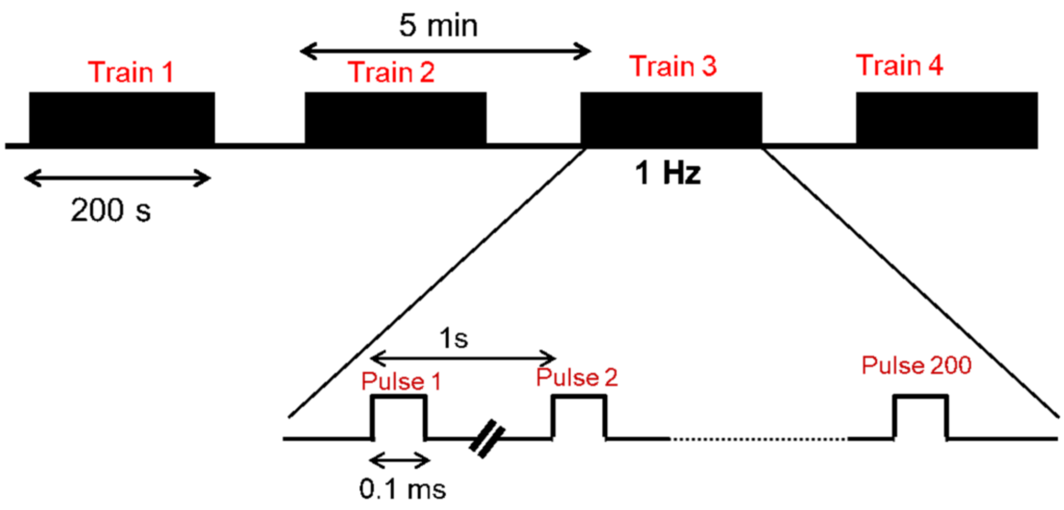

S1. The schematic presentation of stimulation protocol.

Supplementary Fig. 2

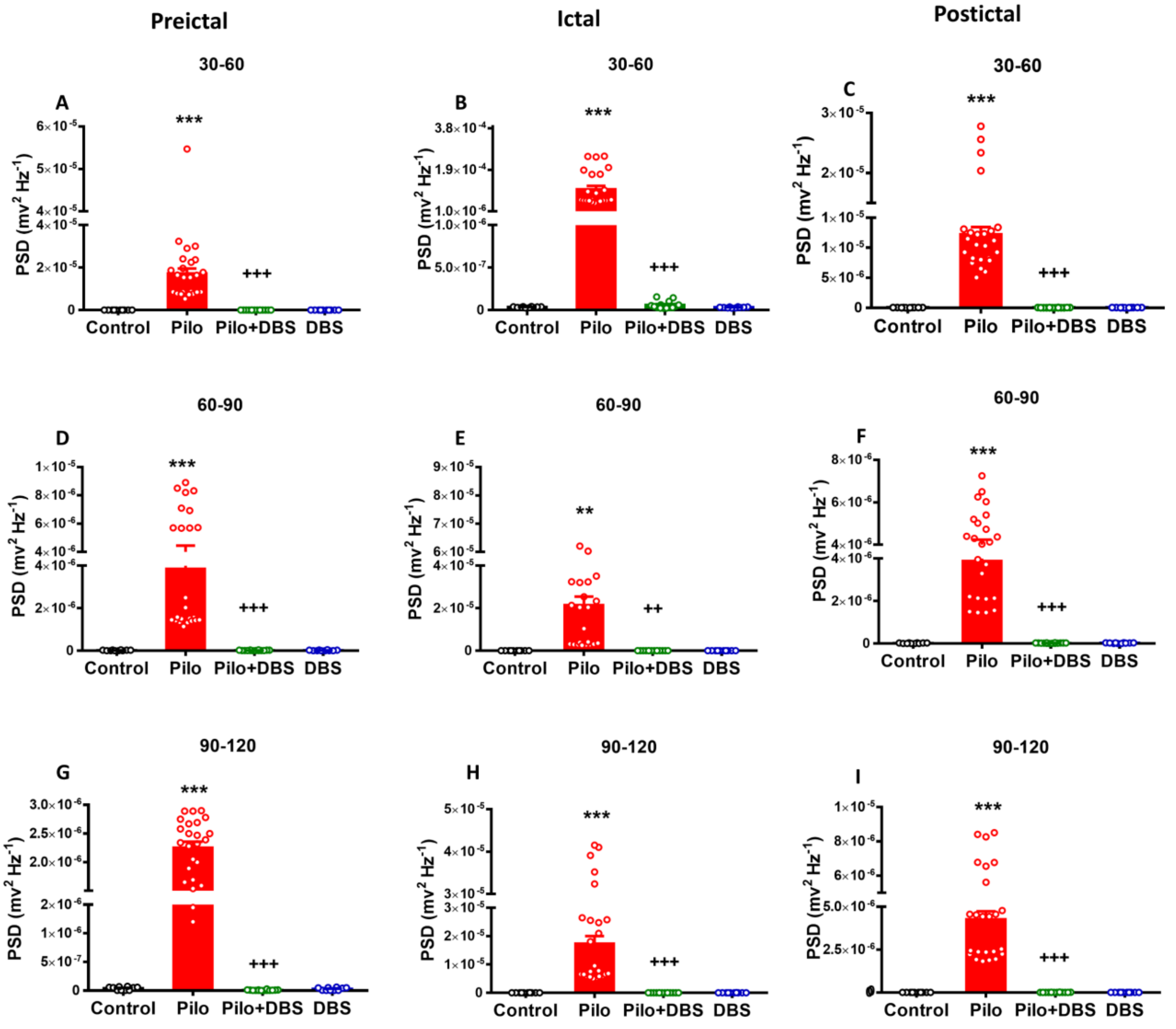

S2. The effect of closed-loop low-frequency DBS on the power spectral density of the gamma sub-bands in hippocampus of epileptic rats in pre-ictal, ictal and post-ictal periods. The power spectral density of slow gamma bands (30-60 Hz) increased in pilocarpine group. Applying DBS significantly decreased these parameters in pre-ictal (A), ictal (B) and post-ictal (C) periods. The same changes were observed for medium gamma bands (60-90 Hz) (D, E and F) and fast gamma bands (90-120 Hz) (G, H and I). Data are presented as means ± SEM. (n = 8). ** $P<0.01$ and *** $P<0.001$ compared to control group. ++ $P<0.01$ and +++ $P<0.001$ compared to pilocarpine (Pilo) group.

Supplementary Fig. 3

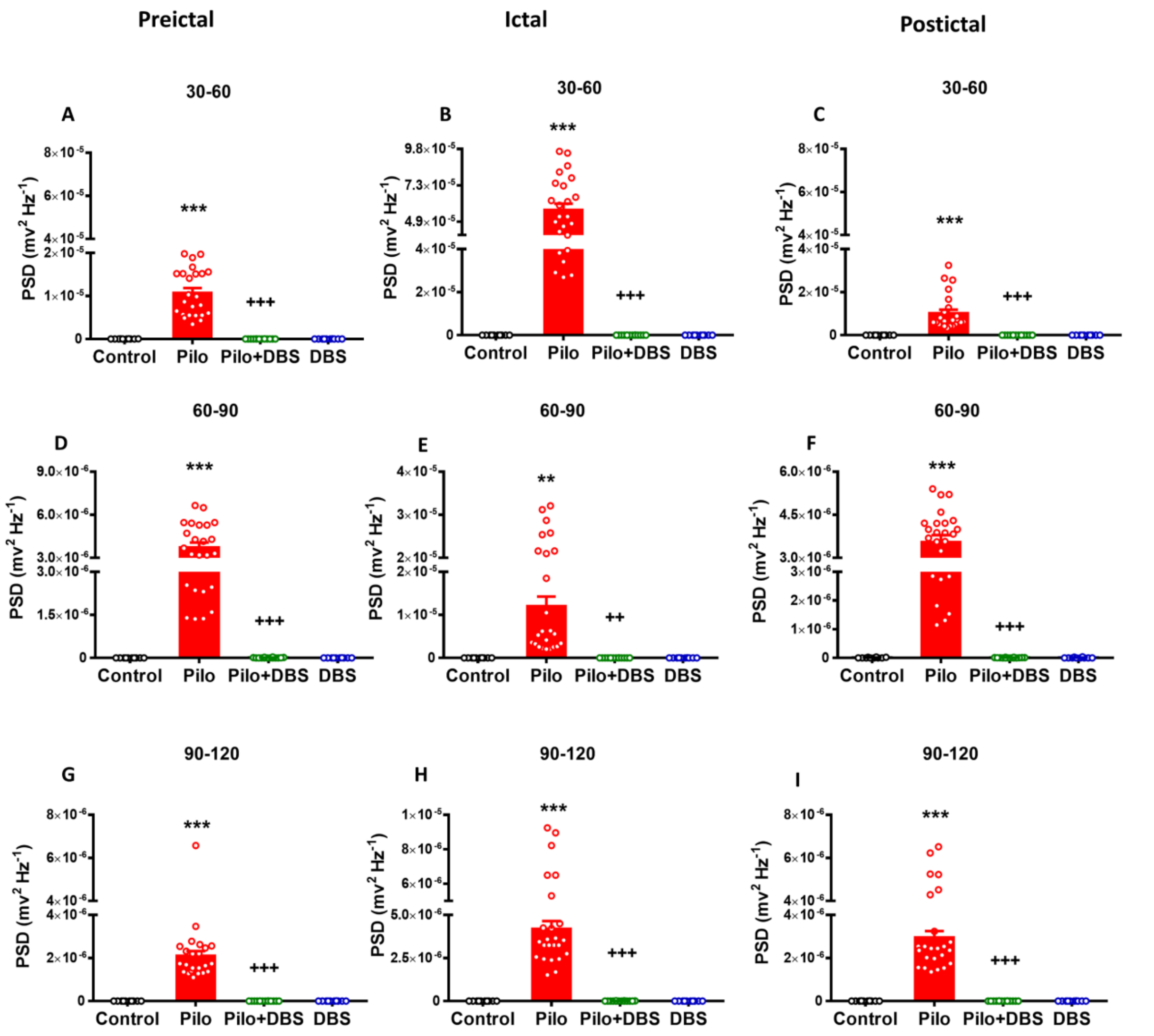

S3. The effect of closed-loop low-frequency DBS on the power spectral density of the gamma sub-bands in mPFC of epileptic rats in pre-ictal, ictal and post-ictal periods. The power spectral density of slow gamma bands (30-60 Hz) increased in pilocarpine group. Applying DBS significantly decreased these parameters in pre-ictal (A), ictal (B) and post-ictal (C) periods. The same changes were observed for medium gamma bands (60-90 Hz) (D, E and F) and fast gamma bands (90-120 Hz) (G, H and I). Data are presented as means ± SEM. (n = 8). ** P<0.01 and *** P<0.001 compared to control group. ++ P<0.01 and +++ P<0.001 compared to Pilo group. Data are presented as means ± SEM. (n = 8). ** P<0.01 and *** P<0.001 compared to control group. ++ P<0.01 and +++ P<0.001 compared to pilocarpine (Pilo) group.

Supplementary Fig.4

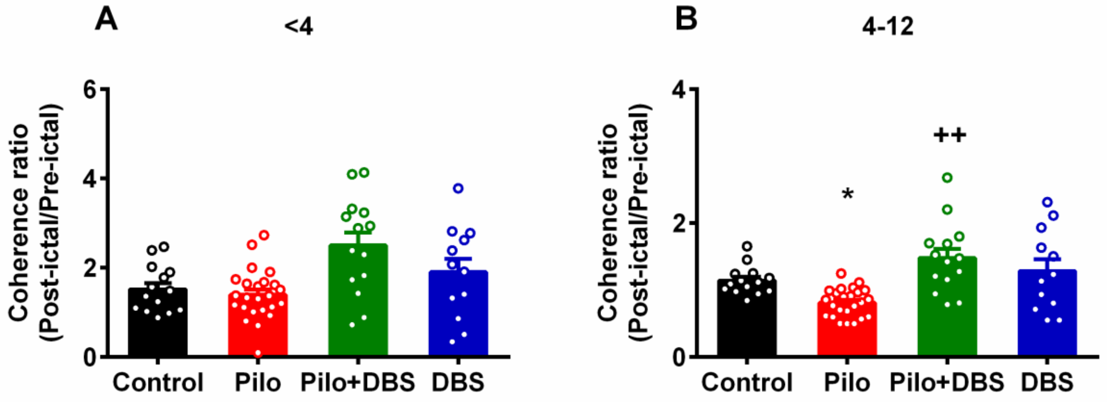

S4. The effect of applying a closed-loop low-frequency DBS on post-ictal/pre-ictal ratio of coherence for delta (A) and theta (B) bands. Applying DBS in Pilo+DBS group restored the change of this ratio for theta band. Data are presented as means ± SEM. (n = 8). * P<0.05 compared to control group. ++ P<0.01 compared to Pilo group.

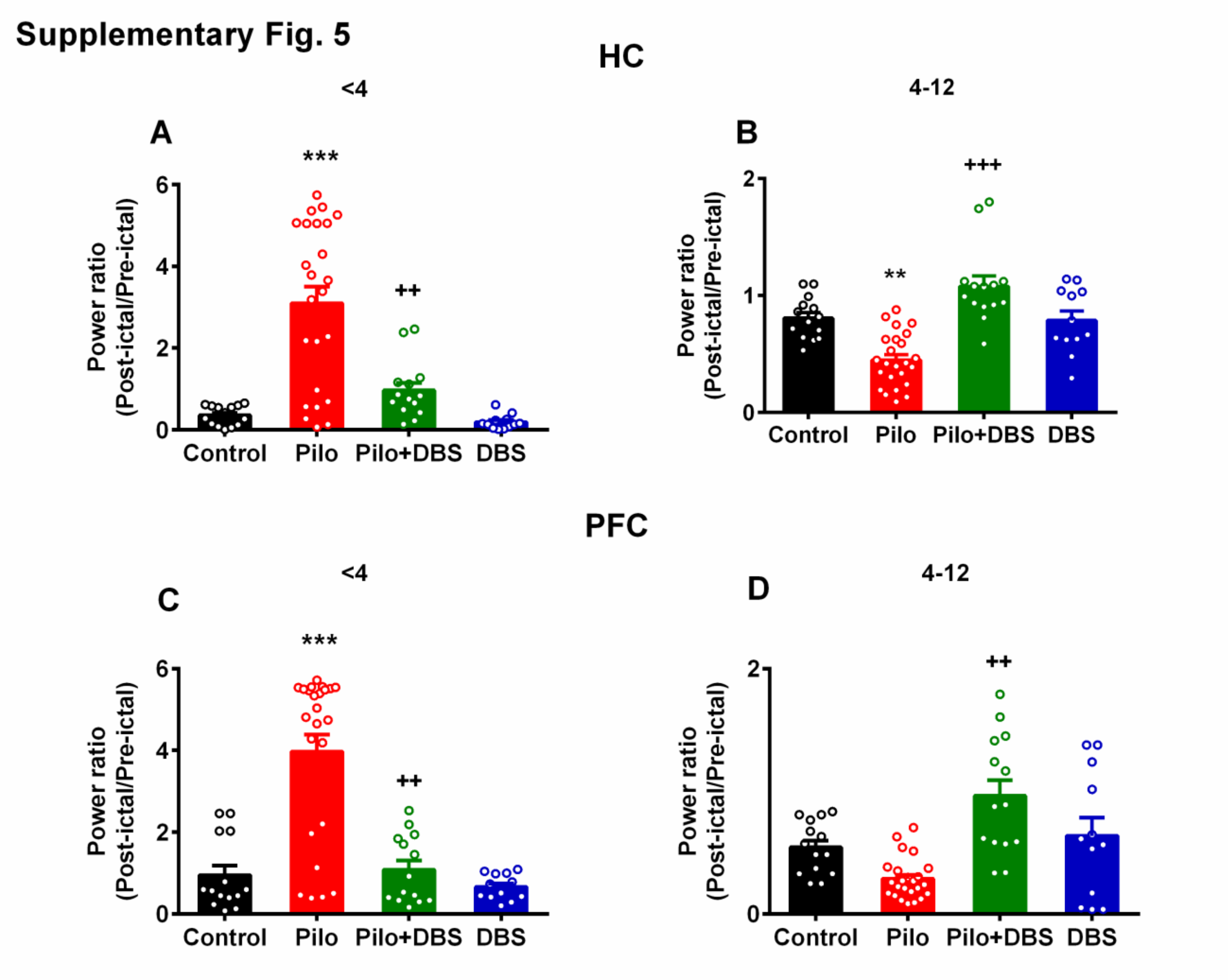

S5. The effect of applying a closed-loop low-frequency DBS on post-ictal/pre-ictal ratio of the power of delta (<4 Hz) and theta (4-12 Hz) bands. The power ratio in delta band increased in pilocarpine (Pilo) group in the hippocampus (HC) (A) and mPFC (C). Applying DBS restored this parameter in both area (A and C). The post-ictal/pre-ictal ratio of the power of theta band (4-12 Hz) decreased in pilocarpine group in the hippocampus (B) and mPFC (D) areas. Applying DBS restored this parameter in both areas (B and D). Data are presented as means ± SEM; (n = 8). ** P<0.01 and *** P<0.001 compared to control group. ++ P<0.01 and +++ P<0.001 compared to pilocarpine (Pilo) group.

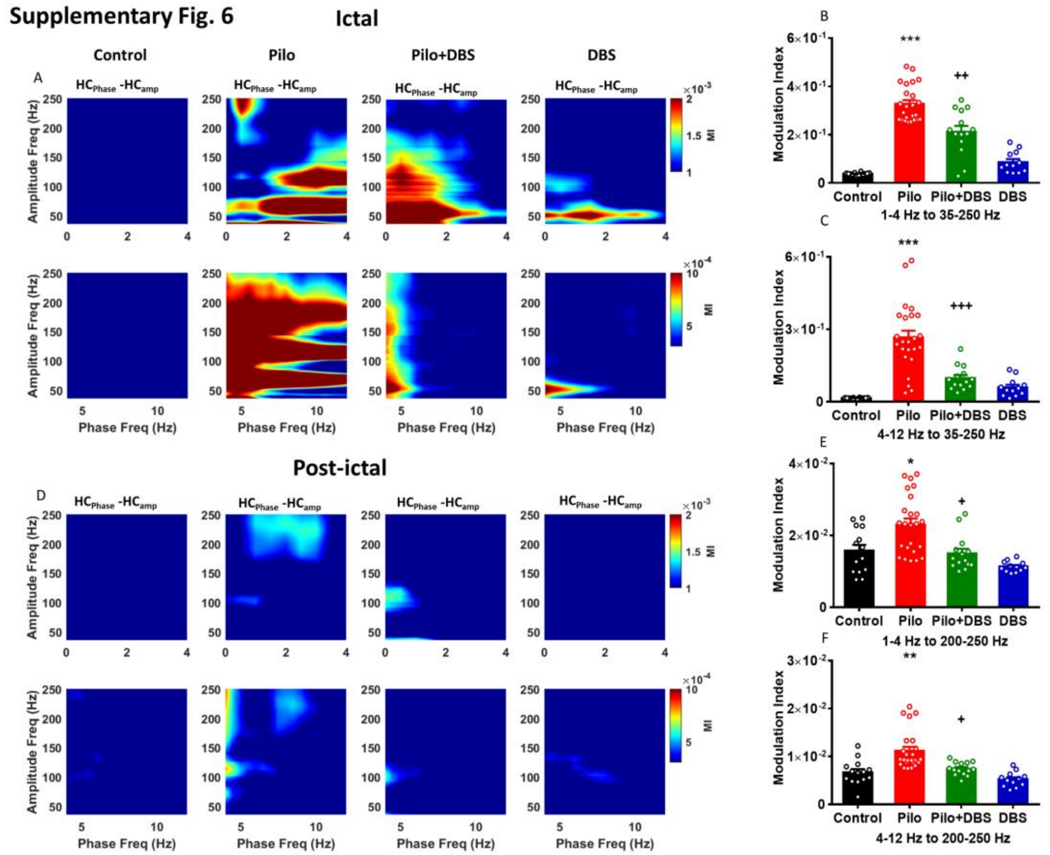

S6. The effect of applying a closed-loop low-frequency DBS on the cross frequency coupling in the hippocampus. Part (A) shows the representative comodulograms of modulation index computed for hippocampal delta (1-4 Hz) and theta (4-12 Hz) phase and gamma amplitude (30-250 Hz) during ictal phase. The bar graphs represent mean values of modulation index for hippocampal delta phase to gamma amplitude (B) and hippocampal theta phase to gamma amplitude (C). Part (D) shows the representative comodulograms of modulation index computed for hippocampal delta (1-4 Hz) and theta (4–12 Hz) phase and gamma (30-250 Hz) amplitude during post-ictal phase. The bar graphs represent mean values of modulation index for hippocampal delta phase to gamma amplitude (E) and hippocampal theta phase to gamma amplitude (F). Data are presented as means ± SEM. (n = 8). * P<0.05; ** P<0.01 and *** P<0.001 compared to control group. + P<0.05; ++ P<0.01 and +++ P<0.001 compared to pilocarpine (Pilo) group.

Supplementary Fig. 7

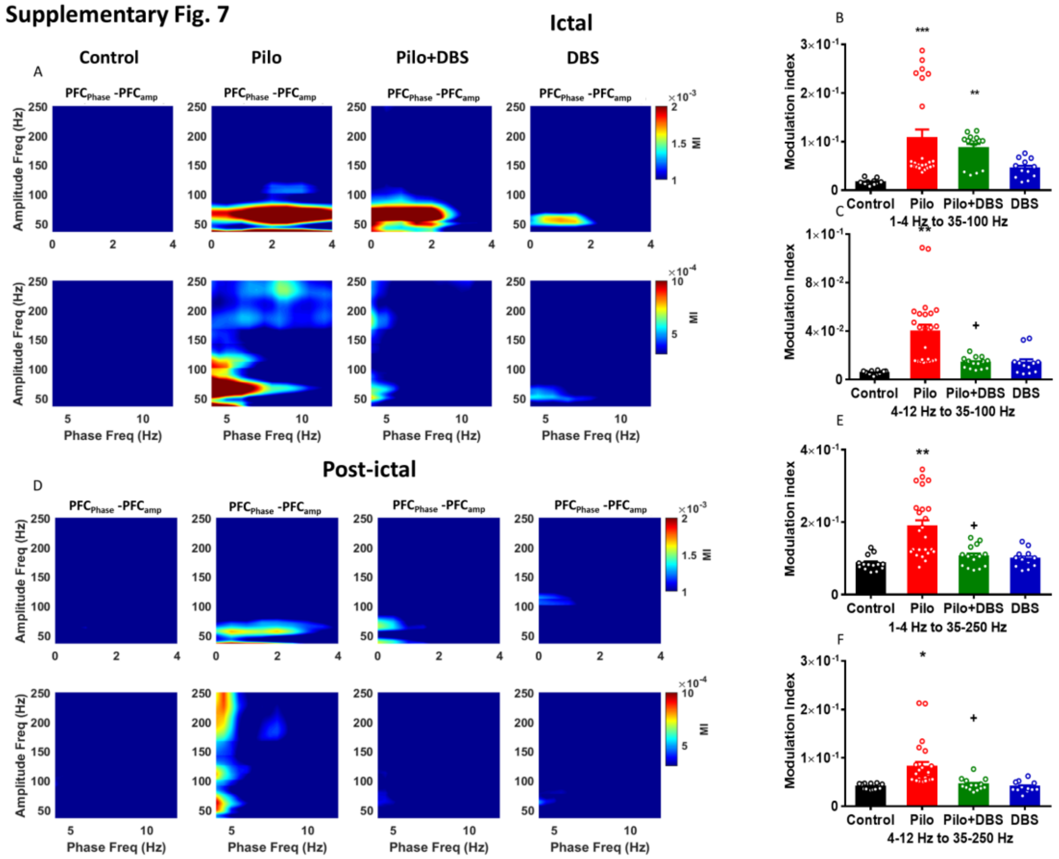

S7. The effect of applying a closed-loop low-frequency DBS on the cross frequency coupling in the mPFC. Part (A) shows the representative comodulograms of modulation index computed for mPFC delta (1-4 Hz) and theta (4-12 Hz) phase and gamma amplitude (30-250 Hz) during ictal phase. The bar graphs represent mean values of modulation index for mPFC delta phase to gamma amplitude (B) and mPFC theta phase to gamma amplitude (C). Part (D) shows the representative comodulograms of modulation index computed for mPFC delta (1-4 Hz) and theta (4–12 Hz) phase and gamma (30-250 Hz) amplitude during post-ictal phase. The bar graphs represent mean values of modulation index for mPFC delta phase to gamma amplitude (E) and mPFC theta phase to gamma amplitude (F). Data are presented as means ± SEM. (n = 8). * $P<0.05$; ** $P<0.01$ and *** $P<0.001$ compared to control group. + $P<0.05$; ++ $P<0.01$ and +++ $P<0.001$ compared to pilocarpine (Pilo) group.
